# Thermal Conductance of the Gold–Water Interface: Implications for Cooling Rates, Melting, and Solidification in Laser Processing of Colloidal Nanoparticles

**DOI:** 10.1021/acs.jpcc.5c06989

**Published:** 2025-12-31

**Authors:** Mikhail I. Arefev, Antonios S. Valavanis, Leonid V. Zhigilei

**Affiliations:** Department of Materials Science and Engineering, 2358 University of Virginia, Charlottesville, Virginia 22904-4745, United States

## Abstract

Thermal conductance at the nanoparticle–liquid interface plays an important role in the heat transfer from colloidal nanoparticles rapidly heated by short-pulse laser irradiation to the surrounding liquid environment. In this study, interfacial thermal conductance is investigated in nonequilibrium molecular dynamics simulations performed for conditions characteristic of laser processing involving transient melting and resolidification of Au nanoparticles in water. The dependence of the Au–water interfacial thermal conductance on the nanoparticle temperature, pressure in the surrounding water, and curvature of the interface is systematically investigated, with a particular focus on the regime in which water adjacent to the hot Au surface is heated up to or above its critical temperature. The formation of a layer of supercritical water strongly affects the heat transfer through a planar interface, while high interfacial curvature enhances conductance, suppresses nanobubble formation, and maintains efficient heat transfer, even at high temperatures. The results of the atomistic simulations are incorporated into a continuum model that couples laser-induced electronic excitation and electron–phonon equilibration in the nanoparticles with heat diffusion in water. Validation of the continuum model against atomistic simulations demonstrates a reliable prediction of nanoparticle temperature evolution and melting onset, while a hybrid atomistic–continuum approach with “implicit” water representation further improves efficiency and avoids finite-size artifacts. Simulations of laser melting and resolidification of 7 and 20-nm nanoparticles predict quenching of the transiently melted nanoparticles within 100 ps (7 nm) to several hundred ps (20 nm), with solidification under deep undercooling producing nanocrystalline structures with a high density of planar defects (twins, stacking faults, and grain boundaries). Thus, beyond advancing the understanding of thermal conductance at the Au–supercritical water interface, the results of this study provide insights into the fundamental mechanisms of the laser-induced modification of nanoparticles in a liquid environment.

## Graphical abstract

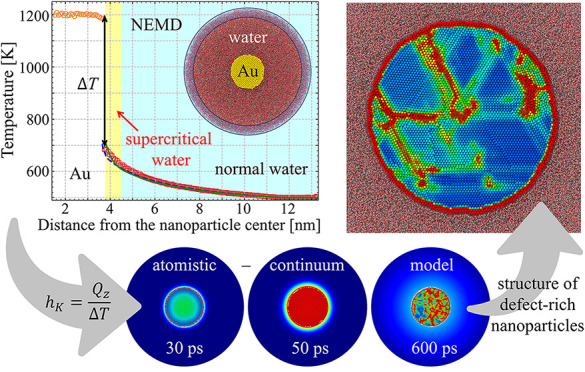

## 1 Introduction

The heat transfer from a colloidal metal nanoparticle rapidly heated by a short laser pulse to the surrounding liquid environment is a process that plays a key role in nanoparticle processing techniques aiming at modification of shape, size, and structure of nanoparticles,^1–6^ as well as in biomedical applications where the laser heating of nanoparticles is used as conduit for precise manipulation of thermal energy at nanoscale.^7–9^ In particular, strongly enhanced catalytic activity of metal nanoparticles produced by laser synthesis in liquids^10–15^ is commonly attributed to the high density of crystal defects,^16,17^ which, in turn, can be related to the extreme cooling rates experienced by the nanoparticles surrounded by a liquid environment.^18–23^ In biomedical applications, the rates and channels of the nanoparticle–liquid energy transfer define the efficiency of the nanoparticle-based photothermal therapy or sensitivity of photoacoustic diagnostics.^24^

In general, heat transfer from a hot nanoparticle to the surrounding liquid is defined by two processes, the heat transfer across the nanoparticle–liquid interface and the diffusional heat dissipation in the liquid environment.^25–28^ The relative importance of the two processes is controlled by the magnitude of the interfacial thermal conductance, *h*_*K*_, and the size of the nanoparticle. The two processes exhibit different scaling with nanoparticle size, with the characteristic time of cooling due to the heat diffusion in the surrounding liquid increasing quadratically with the nanoparticle size, while the time scale of cooling controlled by the interface conductance scales linearly with the size.^25,28^

For one of the most well-studied systems, gold nanoparticles in an aqueous environment, the values of *h*_*K*_ evaluated in time-resolved optical spectroscopy measurements^27,29–31^ cover a broad range from 110 to 450 MWm^–2^ K^–1^, while *h*_*K*_ of 180–225 MWm^–2^ K^–1^ is inferred from photoacoustic signals produced by laser-induced nanobubbles^32^ and *h*_*K*_ of 105 MWm^–2^ K^–1^ is obtained by probing the evolution of lattice expansion in Au nanoparticles in time-resolved X-ray scattering experiments.^33^ The broad range of literature values can be partially attributed to the sensitivity of the interfacial conductance to the type and concentration of surfactants used for stabilization of homogeneous aqueous solutions of Au nanoparticles.^30,31^ Moreover, the values of *h*_*K*_ obtained by ascribing the variation of optical signal in pump–probe spectroscopy solely to the evolution of nanoparticle temperature^27,30,31^ may result in significant overestimation of *h*_*K*_ due the neglect of the contribution from the transient heating of liquid environment to the optical response.^29^ Despite the uncertainty in the values of *h*_*K*_, an estimation of the characteristic time scales of the interfacial thermal conductance and diffusional heat dissipation in the aqueous environment^25–28^ suggests that the two processes make comparable contributions to the cooling of laser-excited Au nanoparticles with sizes in tens of nanometers, while the interfacial conductance plays the dominant role in controlling the rate of cooling for nanoparticles smaller than ten nanometers.

The physical picture of the nanoparticle–liquid heat transfer is further complicated when the temperature of the nanoparticle is sufficiently high to induce phase transformation in a liquid layer adjacent to the nanoparticle surface. The phase transformation can take the form of an explosive boiling^34^ and formation of a nanobubble around a heated nanoparticle,^35–40^ which can significantly slow down the cooling of the nanoparticle.^33–36^ For sufficiently small nanoparticles, the nanobubble formation can be suppressed by the curvature-induced pressure that prevents nucleation of a vapor layer^41,42^ or results in collapse of the transient bubble into a thin layer of supercritical fluid.^20^ Compared to the vapor nanobubble, the compressed supercritical layer is expected to provide a more effective conduit for the heat transfer from a hot nanoparticle to the surrounding liquid, although quantitative data on the thermal conductance of the nanoparticle–supercritical water interface is still lacking.

The complexity of the nanoscale heat transfer from hot nanoparticles to the surrounding liquid calls for multiscale computational analysis of the interplay of various involved processes. At the atomic scale, nonequilibrium molecular dynamics (NEMD) simulations have been used to investigate the dependence of the nanoparticle–liquid heat transfer on nanoparticle size,^43–46^ shape/faceting,^47,48^ strength of the nanoparticle–fluid interaction (wetting),^44–46,49,50^ heat transfer intensity (nanoparticle temperature),^42,51^ polarizability of a metal surface in contact with a polar solvent,^52^ and presence of ligands.^52^ The insights into the mechanisms and channels of heat transfer in the nanoparticle–liquid system obtained in NEMD simulations can potentially contribute to the design of predictive continuum-level models incorporating a description of the interfacial conductance into the heat diffusion equation^27–29,33,53,54^ and/or accounting for the density evolution in the liquid environment triggered by the phase transformations and nanobubble formation.^55–59^ The physical picture provided by NEMD simulations, however, is still fragmented and incomplete, particularly on the heat transfer at high temperatures,^41,42,50,51,60^ when the liquid adjacent to a hot nanoparticle can be heated close to its critical point. Atomistic modeling of the heat transfer in the presence of a layer of supercritical fluid or a nanobubble is particularly challenging and susceptible to artifacts introduced by the small sizes of computational systems and boundary conditions.^50,51,60^

In this work, we perform a systematic investigation of processes controlling the heat transfer from a colloidal Au nanoparticle rapidly heated by a short laser pulse to the surrounding water. The focus of our study is on conditions in which the nanoparticle structure is modified by laser-induced melting, quenching, and resolidification. The analysis of the heat transfer, therefore, is extended to the largely unexplored regime where the liquid adjacent to the nanoparticle surface is transiently heated up to or above the critical temperature of water. The dependence of the Au–water interfacial thermal conductance on the nanoparticle temperature, pressure in the surrounding water, and curvature of the interface is first investigated in a series of NEMD simulations performed with a realistic representation of water. The simulations reveal an important role the formation of a layer of supercritical water plays in defining the heat transfer from the nanoparticle, as well as the strong dependence of the thermal boundary conductance on the size of the nanoparticle, i.e., on the curvature of the interface. The results of MD simulations are then used for the parametrization of a continuum-level model for the nanoparticle–water heat transfer, capable of computationally efficient analysis of the nanoparticle cooling rates realized in laser processing of colloidal nanoparticles. The predictions of the continuum model are verified against the results of molecular dynamics (MD) simulations of laser heating, melting, cooling, and resolidification of 7 and 20-nm Au nanoparticles irradiated in water by 10 ps laser pulses. Finally, a hybrid atomistic-continuum model combining an “implicit” (continuum-level) representation of the water environment with an atomistic MD description of the nanoparticles is designed and applied for the analysis of laser-induced structural modification of nanoparticles undergoing partial or complete melting and resolidification.

## 2 Thermal Conductance of Gold–Water Interface

The investigation of the heat transfer from colloidal Au nanoparticles to the surrounding water is performed in the following steps. First, the ability of the atomistic model of water to reproduce the experimental temperature dependence of thermal conductivity in the temperature range extending to and above the thermodynamic critical point is verified and discussed in Section 2.1. The NEMD simulations are then applied for evaluation of the Au–water interfacial thermal conductance for planar interfaces (Section 2.2) and curved interfaces between Au nanoparticles and the water environment (Section 2.3). The results of the NEMD simulations of thermal conductivity and interfacial thermal conductance are used to design and parametrize continuum-level and hybrid atomistic–continuum models for simulations of laser-induced heating, melting, cooling, and resolidification of colloidal nanoparticles. The formulation of these models, along with the results of the corresponding simulations of laser processing of colloidal nanoparticles, is presented in Section 3.

In this section, the technical details common to the NEMD simulations of thermal conductivity and interfacial conductance are described first, with additional computational parameters specific to individual simulation sets provided in the corresponding subsections.

The MD simulations are performed with LAMMPS.^61^ The interatomic interactions between Au atoms are described by an embedded atom method (EAM) potential^62^ parametrized with focus on high-temperature material properties relevant to the simulations of laser-induced melting and resolidification. In particular, the potential predicts the melting temperature of *T*_m_^EAM^ = 1318 K and enthalpy of melting of Δ*H*_m_^EAM^ = 13.03 kJ/mol, which are close to the corresponding experimental values^63^ of *T*_m_ = 1337 K and Δ*H*_m_ = 12.72 kJ/mol. Note that the effect of the polarizability of metal in contact with a polar solvent, such as water, on the Au–water interfacial thermal conductance has been found to be moderate^64^ and is not considered in the simulations. The water is described with the popular extended simple point charge SPC/E nonflexible model,^65^ which predicts the value of the critical temperature of water, *T*_c_^SPC/E^ = 623 K,^66^ close to the experimental value of 647 K.^67^ This potential has also been shown to provide a good description of water thermal conductivity under atmospheric^68–70^ and high pressure^71^ conditions. The internal rigidity of water molecules is enforced by the SHAKE algorithm.^72^ The long-range Coulomb interactions in water are described with a particle–particle particle-mesh solver.^73^ The integration time step in all simulations was 1 fs.

Water–gold interactions are described by the Lennard–Jones potential defined as a function of the distance between oxygen and gold atoms, while the hydrogen atoms do not interact with the gold atoms. The parameters defining the energy and length scales of the potential are ε = 0.02558 eV and σ = 3.6 Å,^42,74^ chosen to represent normal wetting conditions^75^ with a contact angle of about 25°. A cutoff function^76^ is used to ensure that the oxygen–gold interactions vanish at a cutoff distance of 9 Å. An alternative description of Au–water interaction with the Spohr potential^77^ parametrized based on the adsorption energy of water molecules on a gold surface from thermal programmed desorption experiments^34^ was also considered but not used in the simulations reported in this paper. The Spohr potential predicts a substantially stronger first peak in the water density distribution in the vicinity of a planar Au interface, indicating stronger wetting behavior. As a result, the interfacial conductance at Au temperature of 400 K calculated with the Spohr potential, about 235 MWm^–2^ K^–1^, is approximately double that predicted with the Lennard–Jones potential (see Section 2.2). Note that the fraction of oxidation states on surfaces of colloidal Au nanoparticles in pure water is found to be below 2.15%,^78^ suggesting a negligible effect of surface oxidation (if any)^79^ on the thermal boundary conductance.

### 2.1 Thermal Conductivity of SPC/E Water: From Liquid to Supercritical Fluid

Before applying MD simulations for evaluation of thermal conductance of the Au–water interface, the ability of the SPC/E potential to describe the temperature dependence of thermal conductivity of water *k*(*T*) is tested at a constant critical point pressure of 22 MPa. The thermal conductivity is evaluated using the NEMD technique, where a temperature gradient ∇*T* is created across an MD computational cell by defining heat bath regions, where constant temperatures are maintained during the simulation. Once the steady state is reached in the system, the heat flux *Q* is calculated in the direction of the temperature gradient, and Fourier’s law is applied to obtain thermal conductivity, *k* = −*Q*/∇*T*.

The calculations of thermal conductivity of SPC/E water are performed for a system with initial (at 300 K and 22 MPa) dimensions of 3.56 × 3.56 × 21.2 nm^3^ and periodic boundary conditions applied in all directions. Two 3-nm-thick slices of the system in the direction normal to the *z*-axis are defined as hot and cold heat bath regions, with constant temperatures in these regions maintained using Langevin thermostats^80^ with a damping time of 0.1 ps. The separation between the two heat bath regions is equal to half of the size of the system in the *z* direction, so that two temperature gradients with opposite direction of the heat flow are established in the system. The simulations are performed for systems with different average temperatures, and, for each average temperature, the hot and cold bath regions are maintained at temperatures 10 K above and below the average, respectively. Each system is first equilibrated at a constant pressure of 22 MPa by applying the Berendsen barostat^81^ acting only in the *z* direction with a damping parameter of 1 ps for 100 ps. Once the steady state temperature profiles and the constant level of pressure are established, the simulations are continued under constant volume conditions for an additional 1 ns, with the last 500 ps used for calculation of the time-averaged temperature profiles *T*(*z*) and heat flux *Q*_*z*_. The latter is calculated from the absolute values of slopes of the cumulative energy addition/removal by the cold and hot thermostats, averaged between the two, and divided by the area of the (*x*, *y*) cross-section of the system. Two values of the temperature gradient d*T*/d*z* are then obtained by fitting the two linear parts of the temperature profiles established between the heat baths. Finally, the values of the thermal conductivity are obtained as *k* = −*Q*_*z*_/(d*T*/d*z*).

The results of the calculations are shown in Figure 1, where the predictions of NEMD are compared with an experimental curve plotted for the same pressure of 22 MPa. The overestimation of room temperature thermal conductivity by about 20% is consistent with earlier studies performed at 1 atm pressure with the same potential,^68–70^ where the values ranging from 0.73 to 0.94 W m^–1^ K^–1^ are reported. The observation that the room temperature value of 0.86 W m^–1^ K^–1^ obtained in the present study at 22 MPa is within the range of values obtained at 1 atm reflects a relatively weak pressure dependence of the thermal conductivity of the SPC/E water.^71^ The latter is also consistent with the behavior of real water, where the room temperature thermal conductivity increases by about 8% upon pressure increase from 1 atm to 100 MPa.^82^

**Figure 1:**
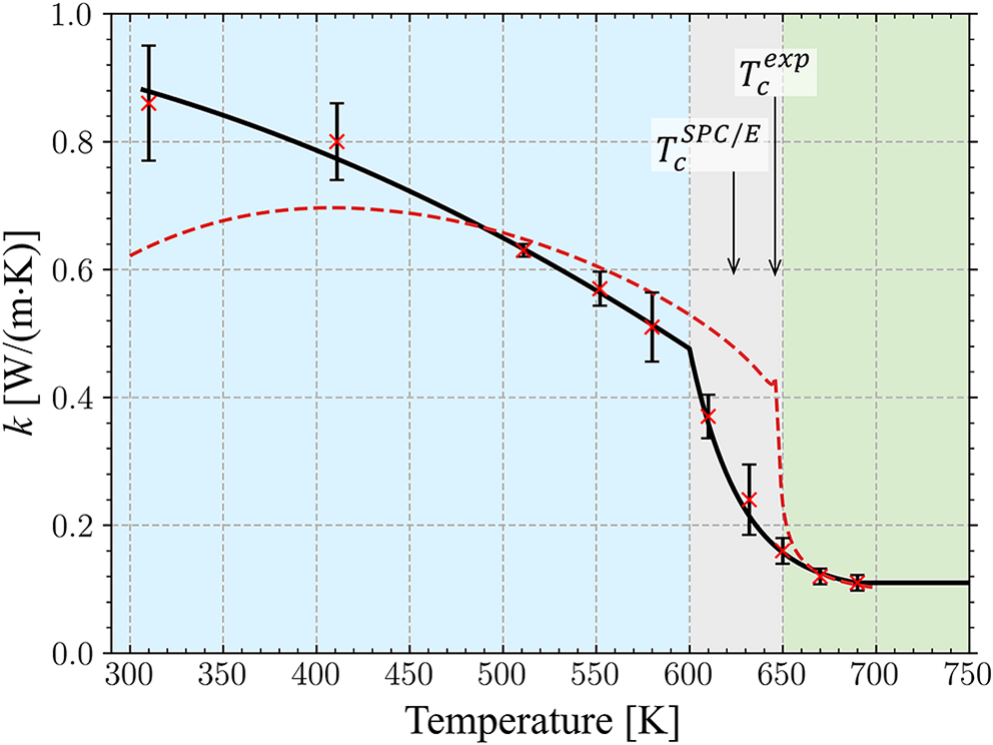
Thermal conductivity of SPC/E water obtained in a set of NEMD simulations performed at a constant pressure of 22 MPa (red crosses). The error bars are evaluated as a standard deviation based on the magnitudes of heat fluxes *Q*_*z*_ of cold and hot thermostats and two values of d*T*/d*z*. The background color marks the regions of normal water (light blue), transition to the supercritical state (light gray), and supercritical water (light green). The black line is a piecewise fit to the data points to a quadratic polynomial for *T* ≤ 600 K, an exponential drop for 600 < *T* < 690 K, and a constant level of 0.11 W m^–1^ K^–1^ for *T* ≥ 690 K. The dashed red line is the experimental data provided by the International Association for the Properties of Water and Steam (IAPWS) for 22.065 MPa.^67,83^ The vertical arrows indicate the experimental critical temperature of water and the corresponding value predicted by the SPC/E potential.

At higher temperatures, the discrepancy between computational and experimental results decreases, with both data sets exhibiting a sharp drop upon the transition to the supercritical state and saturation at almost the same level in the supercritical state. The transition to the supercritical region is shifted to lower temperatures with respect to the experimental curve, reflecting the lower critical temperature of the SPC/E water, *T*_c_^SPC/E^ = 623 K^66^ versus *T*_c_^exp^ = 647 K.^67^ Note that earlier calculations exploring the thermal conductivity of water in the vicinity of *T*_c_ reported an opposite trend of SPC/E data point being shifted slightly to higher temperatures with respect to the experimental curve at 19 MPa.^71^ Overall, despite the moderate overestimation of thermal conductivity at room temperature and the small discrepancy in the critical point, the results plotted in Figure 1 demonstrate a remarkable ability of the SPC/E potential to provide a reliable quantitative description of water thermal conductivity in a broad temperature range, from 300 K to the supercritical state.

### 2.2 Thermal Conductance of Planar Au–Water Interface

The calculation of thermal conductance of the planar Au–water interface is performed with the NEMD setup similar to that discussed in the previous section, except that the initial SPC/E water system is modified by inserting a layer of Au in the center of the system and removing water molecules overlapping with the gold layer, Figure 2. The orientation of (001) planes in the Au layer is perpendicular to the *z*-axis, and the thickness of the Au layer corresponds to 8 face-centered cubic (fcc) unit cells. The initial (at 300 K and 22 MPa) dimensions of the computational system are 3.56 nm × 3.56 nm × 66.4 nm, with periodic boundary conditions applied in all directions. The temperature gradient in the combined Au–water system is established by applying Langevin thermostats with a damping time of 0.1 ps^80^ to a 6-nm-wide cold heat bath region (the two parts of the heat bath in Figure 2 are connected with each other by periodic boundary conditions) and maintaining the temperature of the Au layer at a higher temperature. To indirectly account for a fast heat transfer in Au due to the electron heat conduction and to avoid the formation of a temperature gradient within the Au layer, the Au layer is split into 5.5 Å slices, and individual Langevin thermostats are applied to each slice. The pressure was maintained at approximately 22 MPa with Berendsen barostat^81^ with a damping parameter of 1 ps acting only in the *z* direction. To avoid a possible extra contribution to the total pressure of the system due to thermal expansion of Au, we adjust the lateral size of the computational cell according to the thermal expansion coefficient calculated for the EAM potential and only apply the barostat to water molecules.

**Figure 2:**
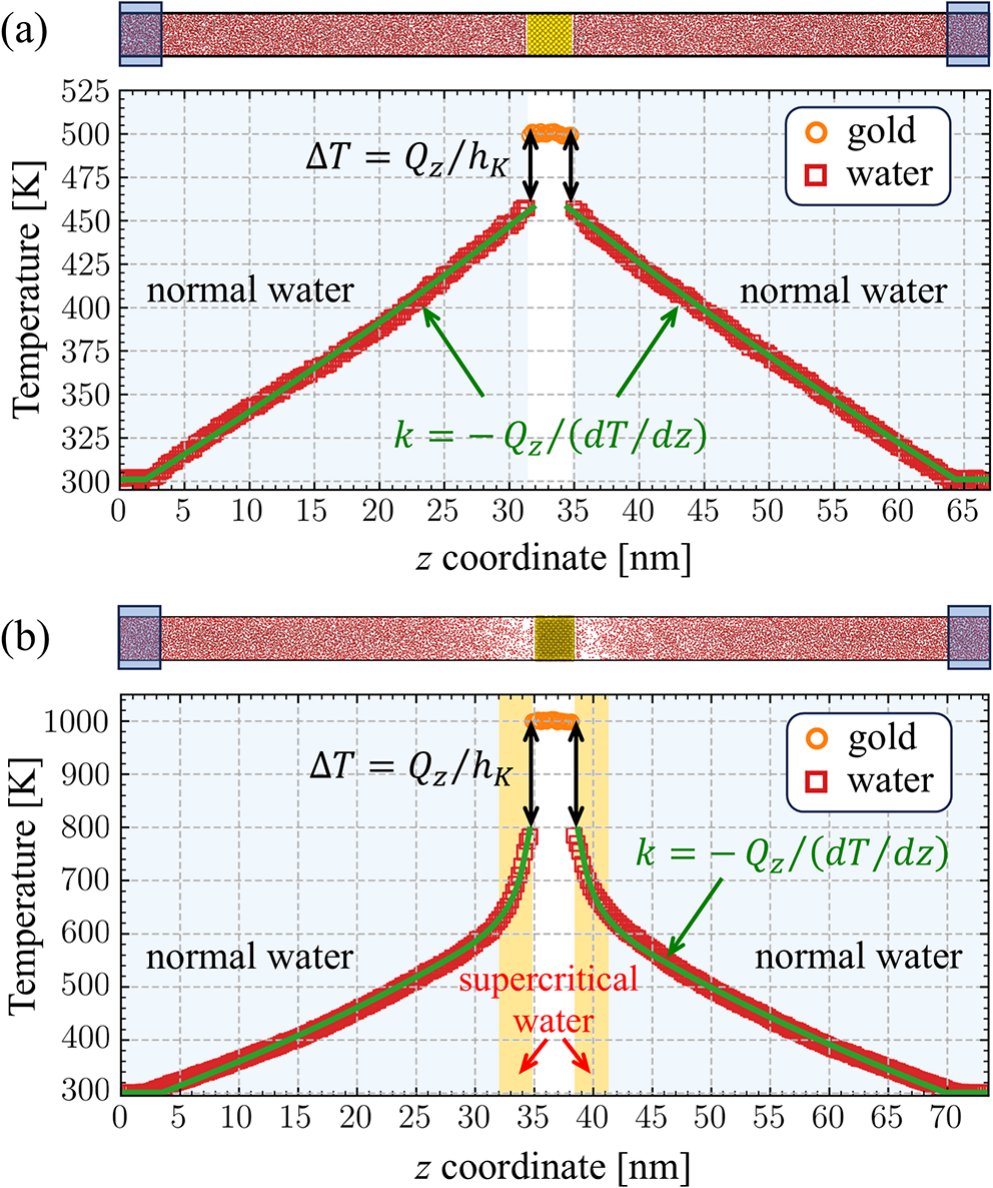
Computational setup for calculation of the interfacial thermal conductance *h*_*K*_ of a planar water–Au interface. Atomic configurations and temperature profiles are shown for steady-state conditions established in NEMD simulations where the temperature of Au is maintained at 500 (a) and 1000 K (b). The temperature of water in the heat bath regions (marked by blue rectangles in the snapshots) is maintained at 300 K. The pressure in water is maintained at 22 MPa. The temperature distributions predicted in the NEMD simulations are shown by orange circles for Au and red squares for water. The black double-headed arrows mark the temperature jump Δ*T* at the Au–water interface. The thermal boundary conductance at the interface, *h*_*K*_, is calculated based on Δ*T* and the steady state heat flux in the *z* direction, *Q*_*z*_, as *h*_*K*_ = *Q*_*z*_/Δ*T*. The green lines are the profiles calculated with the continuum heat diffusion model using *k*(*T*) and *h*_*K*_ obtained in the NEMD simulations. In the snapshots, gold, oxygen, and hydrogen atoms are colored orange, red, and light gray, respectively.

Two series of NEMD simulations were performed with water temperature in the cold bath region, *T*_w_^HB^, maintained at 300 K in one series and at 500 K in another series of simulations. The first series of simulations was performed for 11 temperatures of Au, *T*_Au_, ranging from 400 to 1400 K, with 100 K intervals. The second series consisted of 9 simulations performed for Au temperatures at and above 600 K. The comparison of the results obtained at two different cold bath temperatures makes it possible to evaluate the effect of the magnitude of the heat flux on the value of *h*_*k*_. For each set of hot and cold bath temperatures (*T*_Au_ and *T*_w_^HB^), the NEMD simulation is first performed for 3.1 ns to ensure that steady-state heat transfer conditions, when the energy added by the hot thermostat equals the energy removed by the cold thermostat, are established. The simulation is then continued for an additional 300 ps, with the final temperature and density profiles calculated by averaging over data collected every 100 steps (0.1 ps) of this part of the simulation. The value of the heat flux along the *z* direction, *Q*_*z*_, is calculated from the slopes of cumulative energy changes recorded for the cold and hot thermostats. The absolute values of the rates of energy addition and removal are averaged and divided by the area of the (*x*, *y*) cross-section of the system, yielding the value of *Q*_*z*_. Examples of the steady state temperature profiles are shown in Figure 2a,b for the same temperature of the cold heat bath, *T*_w_^HB^ = 300 K, and two temperatures of Au, *T*_Au_ = 500 K and *T*_Au_ = 1000 K. From the temperature distributions, the average of the temperature jumps Δ*T* at the two Au–water interfaces is evaluated, and the thermal boundary conductance is calculated as *h*_*K*_ = *Q*_*z*_/Δ*T*.

The results of the calculations are shown in Figure 3. The values of *h*_*K*_ decrease with increasing temperature of gold, *T*_Au_, and exhibit signs of saturation at *T*_Au_ > 1200 K (Figure 3a). The values of *h*_*K*_ obtained with a higher *T*_w_^HB^ of 500 K are consistently lower than those calculated in simulations performed with *T*_w_^HB^ = 300 K. This observation can be attributed to the higher temperature of water at the interface produced for the same *T*_Au_ at a higher *T*_w_^HB^. Indeed, when the thermal boundary conductance is plotted as a function of water temperature in the close vicinity of the interface, Figure 3c, the results of the two sets of simulations merge into a single trend. The higher temperature of water at *T*_w_^HB^ = 500 K leads to the reduction of the water density near the interface and the heat flux across the interface, leading to the decrease in *h*_*K*_. The dependence of *h*_*K*_ on the heat flux is consistent with earlier NEMD simulations for silicon–water interfaces,^84^ where a similar trend was observed. The observation of *h*_*K*_ decreasing with increasing temperature, however, can be contrasted with an opposite trend observed experimentally for various solid–solid interfaces,^85^ as well as in simulations of solid–liquid interfaces.^84,86,87^ We note, though, that these earlier simulations were performed under conditions of constant volume, when the increase in temperature is accompanied by an increase in pressure, which is likely to be the main factor leading to the increase in the thermal boundary conductance.^84,88^ In this study, a constant pressure of 22 MPa is maintained, the system is allowed to expand, and the liquid density decreases in the vicinity of the interface with a hot metal surface. As discussed below, the decrease in the density of the liquid near the interface is the key factor defining the drop in thermal boundary conductance.

**Figure 3:**
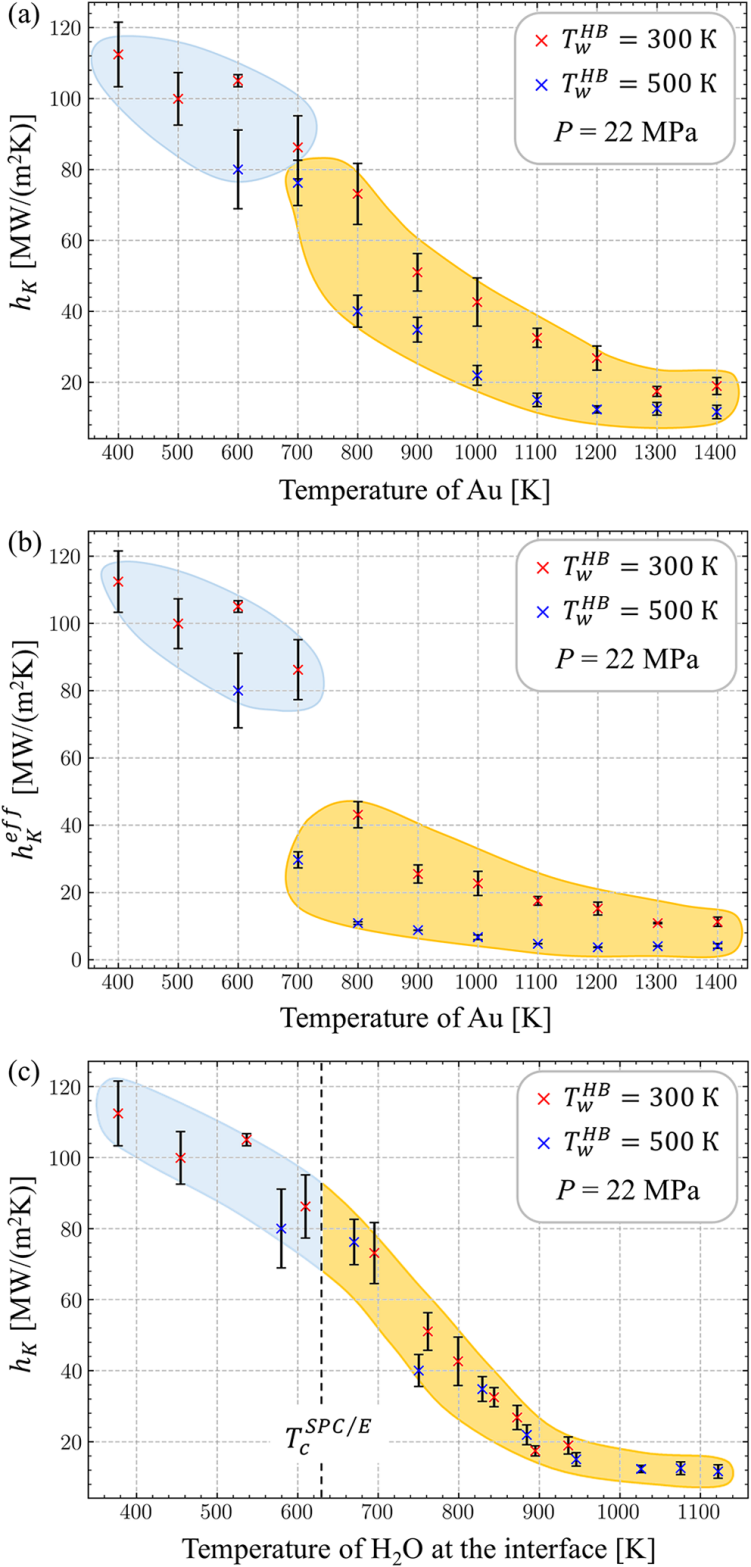
Values of thermal boundary conductance *h*_*K*_ calculated for a planar water–gold interface in NEMD simulations performed with a setup shown in Figure 2. The simulations are performed at different temperatures of Au and two temperatures of water heat bath: 300 K (red symbols) and 500 K (blue symbols). The error bars are calculated as the standard deviation of *h*_*K*_ based on two values of *Q*_*z*_ and Δ*T*. The values of *h*_*K*_ are plotted as a function of temperature of Au in (a) and temperature of water averaged over a 0.4-nm layer adjacent to the Au–water interface in (c). In (b), the effective/combined thermal conductance *h*_*K*_^eff^ of the Au–water interface and the supercritical water layer is plotted as a function of Au temperature. The blue and yellow areas schematically mark the regions below and above the threshold for supercritical water layer formation, respectively.

Two regions can be distinguished in the dependence of *h*_*K*_ on *T*_Au_ plotted in Figure 3a, a low-temperature region (marked by blue color), where *h*_*K*_ gradually decreases from 110 to about 80 MWm^–2^ K^–1^ with *T*_Au_ increasing from 400 to 700 K, and a high-temperature region (marked by yellow color), where *h*_*K*_ drops sharply to about 40 MWm^–2^ K^–1^ and further decreases to 10–20 MWm^–2^ K^–1^ as *T*_Au_ exceeds 1200 K. The transition from the first to the second region corresponds to the formation of a layer of supercritical water, where the temperature of water is above *T*_c_^SPC/E^, as can be seen from Figure 3c, where *h*_*K*_ is plotted as a function of temperature of water averaged over a 0.4-nm layer adjacent to the Au–water interface. For *T*_w_^HB^ = 500 K, the supercritical layer is observed starting from *T*_Au_ = 700 K, when its thickness is about 8 nm. The thickness of the supercritical layer gradually increases with increasing *T*_Au_ and reaches about 23 nm at *T*_Au_ = 1400 K. As the supercritical layer expands, the value of *h*_*K*_ decreases from 76 MWm^–2^ K^–1^ at *T*_Au_ = 700 K to about 12 MWm^–2^ K^–1^ at *T*_Au_ = 1200 K, and remains at approximately the same level upon further increase of *T*_Au_ to 1300 and 1400 K. For *T*_w_^HB^ = 300 K, the presence of the cold heat bath located only 30 nm away from the Au–water interface suppresses the expansion of the supercritical layer. In this series of simulations, the supercritical layer with a thickness of about 2 nm appears only at *T*_Au_ = 800 K and expands to about 4 nm at *T*_Au_ = 1400 K. The corresponding drop in *h*_*K*_ is from 73 MWm^–2^ K^–1^ at *T*_Au_ = 800 K to the lowest value of 17 MWm^–2^ K^–1^ at *T*_Au_ = 1300 K.

Since the pressure in the simulations is maintained at the critical point level of 22 MPa, the interfacial water layer does not evolve into a vapor bubble with a well-defined vapor–liquid interface even when the temperature of the Au surface is twice as high as the critical temperature of the model water. Rather, the density of water varies gradually with distance from the interface (e.g., see snapshot in Figure 2b) and does not exhibit jumps in density and temperature profiles characteristic of the liquid–vapor interface.^89^ Since the definition of the temperature jump Δ*T* at the water–Au interface can become ambiguous when a supercritical layer is present, it is instructive to consider a combined/effective conductance of the Au–water interface together with that of the supercritical layer. We define this effective thermal conductance as *h*_*K*_^eff^ = *Q*_*z*_/(*T*_Au_ – *T*_c_^SPC/E^) when the supercritical layer is present and *h*_*K*_^eff^ = *h*_*K*_ in the absence of the supercritical layer. As can be seen in Figure 3b, *h*_*K*_^eff^ exhibits a pronounced drop upon the formation of the low-density supercritical layer, highlighting the threshold-like dependence of the efficiency of the heat transfer from Au to water on the thermodynamic state of water in the vicinity of the interface, Figure 3c. Note that the high-temperature effective conductance saturates at a level of about 4 MWm^–2^ K^–1^ in a simulation performed with *T*_w_^HB^ = 500 K, where the thickness of the supercritical layer reaches tens of nanometers. This level of thermal conductance is still substantially higher than that characteristic of solid–vapor interfaces.^90–92^

As a check of the self-consistency of the temperature dependence of the thermal conductivity of SPC/E water, *k*(*T*), and the values of the thermal boundary conductance, *h*_*K*_(*T*_Au_,*T*_w_^HB^), the temperature profiles predicted in NEMD simulations are reproduced with a continuum heat diffusion equation. The equation is solved with boundary conditions mimicking those implemented in NEMD simulations, i.e., *T*_w_ = *T*_w_^HB^ at the inner boundary of the heat bath region and *T*_w_ = *T*_Au_ – *Q*_*z*_/*h*_*K*_ at the Au–water interface. The value of *Q*_*z*_ is the steady state heat flux determined in NEMD for a particular set of *T*_Au_ and *T*_w_^HB^. The results of the calculations are exemplified for the two regimes of heat transfer, below and above the threshold for the formation of a supercritical water layer, by the green curves in Figure 2. In both regimes, the continuum solutions reproduce well the results of NEMD simulations, including the steepening of the temperature profile within the supercritical region in Figure 2b. The latter reflects the sharp drop in thermal conductivity upon the transition to the supercritical state that can be seen in Figure 1.

### 2.3 Thermal Conductance of Au Nanoparticle–Water Interface

The conductance of curved interfaces between Au nanoparticles and a water environment is investigated in the NEMD setup illustrated in Figure 4. The simulations are performed for 7- and 20-nm spherical Au nanoparticles surrounded by water. The water environment is represented by an 8.5-nm-thick spherical shell of water surrounding the particle. The systems consist of 10,606 Au atoms and 230,948 water molecules in simulations of 7-nm nanoparticles and 247,413 Au atoms and 692,325 water molecules in simulations of 20-nm nanoparticles. The temperature gradient is established by maintaining constant temperatures of the Au nanoparticle and an outer layer of the water shell with a thickness of δ_HB_ = 1 nm using Langevin thermostats. Similar to the planar interfaces discussed in Section 2.2, several concentric shell-like layers of Au nanoparticles are thermostated separately to prevent the formation of temperature gradients within the nanoparticles. The thickness of the concentric layers is chosen so that they contain approximately the same number of Au atoms. The number of thermostats applied to 7- and 20-nm nanoparticles is four and nine, respectively. Most of the NEMD simulations are performed for two temperatures of the water heat bath region, *T*_w_^HB^ = 300 K and *T*_w_^HB^ = 500 K, while the temperature of Au nanoparticles, *T*_Au_, is varied from 400 to 1400 K with 100 K intervals. An additional simulation is also performed at *T*_w_^HB^ = 400 K and *T*_Au_ = 900 K. By varying *T*_Au_ and *T*_w_^HB^ in the steady-state heat-transfer simulations, we evaluate the interfacial thermal conductance over a range of local temperature gradients established near the nanoparticles. This approach enables direct mapping of the steady-state results to the transient heat-transfer conditions characteristic of the laser–nanoparticle interactions discussed in Section 3.

**Figure 4:**
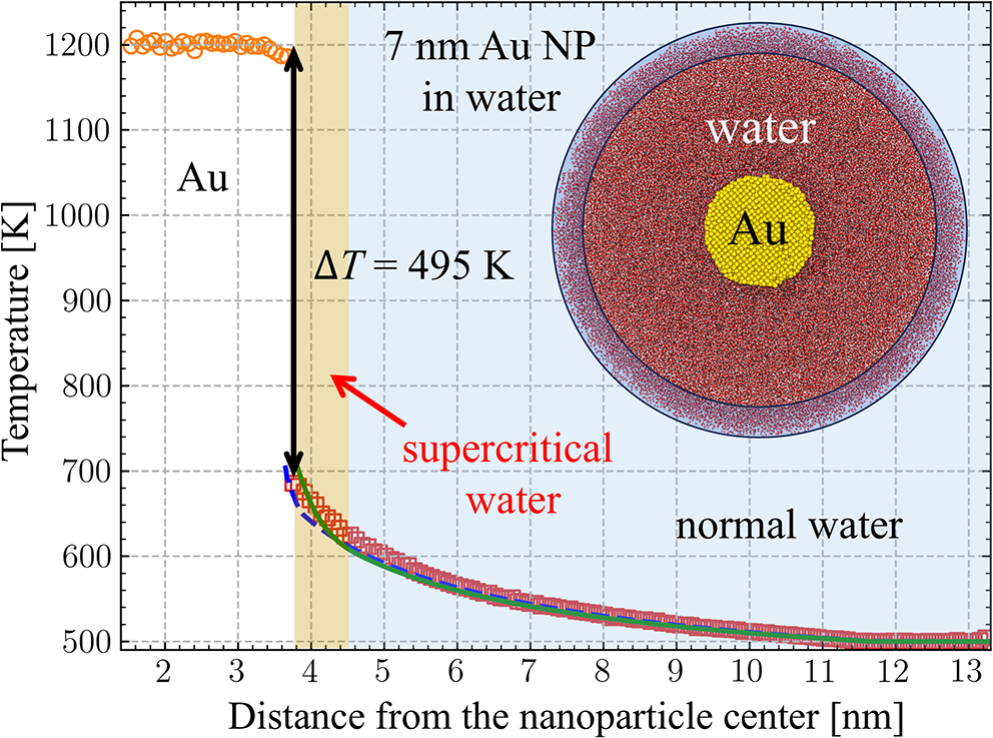
Computational setup for calculation of the interfacial conductance *h*_*K*_ of an interface between a gold nanoparticle and water. A cross-section of the computational system is shown in the inset. A steady-state temperature profile is plotted for a simulation where the temperature of a 7-nm Au nanoparticle is maintained at 1200 K, while the temperature of water in the heat bath region (shown as the blue semitransparent shell in the snapshot) located 8 nm away from the surface of the nanoparticle is maintained at 500 K. The pressure in water is maintained at 22 MPa. The temperature distribution predicted in the NEMD simulation is shown by orange circles for Au and red squares for water. The black double-headed arrow marks the temperature jump Δ*T* at the Au–water interface. The thermal boundary conductance at the interface, *h*_*K*_, is calculated based on Δ*T* and the steady-state heat flux at the interface, *Q*_r_^int^, as *h*_*K*_ = *Q*_r_^int^/Δ*T*. The solid green and dashed blue lines show results of continuum model calculations with the temperature dependence of water thermal conductivity *k*(*T*) predicted for the SPC/E model in NEMD simulations (black line in Figure 1) and experimental data for water (red dashed line in Figure 1),^67,83^ respectively. The conductance *h*_*K*_ obtained in the NEMD is used in both sets of continuum simulations. In the snapshot, gold, oxygen, and hydrogen atoms are colored orange, brown, and light gray, respectively.

To compensate for the surface tension generated by the outer boundary of the water shell and to enable pressure control in the computational cell, an imaginary dynamic spherical wall is applied at the outer boundary. The spherical wall interacts with water via a Lennard–Jones potential defined as a function of the radial distance between the oxygen atoms and the wall. The energy and length parameters of the oxygen–wall interaction, ε_w_ = 6.74 meV and σ_w_ = 3.17 Å, are chosen to mimic the effect of the interaction of water outside the computational cell with atoms in the vicinity of the cell boundary. The pressure in water is controlled by adjusting the radius of the wall for each pair of *T*_Au_ and *T*_w_^HB^.

After the target pressure is established for a given set of *T*_Au_ and *T*_w_^HB^, the simulation is continued to accumulate data for temperature and density profiles, as well as the radial heat flux at the interface *Q*_r_^int^. The latter relies on the definition of radius of the spherical interface since, in contrast to the heat flux through a planar interface considered in Section 2.2, the heat flux decreases with increasing radial location of a spherical cross-section of the system due to the expanding surface area. The radius of the Au–water spherical interface, *R*, is assumed to coincide with the radius of the nanoparticle, with the latter obtained by fitting of the time-averaged Au density profile with a logistic function and finding the radius where the function is equal to 0.5.

The simulation time required for the establishment of steady-state temperature profiles is about 2 ns, and the results of the calculations are illustrated in Figure 4 for a 7-nm Au nanoparticle with *T*_*Au*_ = 1200 K and *T*_w_^HB^ = 500 K. The temperature jump at the interface, Δ*T* = *T*_Au_ – *T*_w_^int^, is defined as the difference between the average temperature of the nanoparticle *T*_Au_ and the temperature of water at the interface *T*_w_^int^. The latter is obtained by linear fitting of six points of the water temperature profile adjacent to the interface (corresponds to an approximately 0.4-nm-thick spherical layer) and extrapolating the fit to the interface position. Note that the solution of the continuum equation shown by the green line in Figure 4 does not account for the local compression produced by the interface between the supercritical and normal water (see discussion below) and cannot be used for the reliable prediction of *T*_w_^int^. The thermal boundary conductance at the interface, *h*_*K*_, is then calculated for a given set of *T*_Au_ and *T*_w_^HB^ as *h*_*K*_(*T*_Au_, *T*_w_^HB^) = *Q*_r_^int^/Δ*T*.

Similar to systems with planar interfaces, the temperature profiles predicted in NEMD simulations are reproduced with a continuum steady-state heat diffusion equation formulated, in this case, in spherical coordinates. The results of the calculations are exemplified by the green line in Figure 4. The solution of the continuum model describes the NEMD results rather well, with deviations at high temperatures related to the Laplace pressure produced by the high-curvature interface between supercritical and normal water. Note that the agreement between the continuum solution of the heat diffusion equation and NEMD results is better in the case of planar Au–water interfaces (Figure 2), where the curvature-related effects are absent. Interestingly, an alternative parametrization of the heat diffusion equation using the experimental IAWPS data for *k*(*T*) at 22.065 MPa,^67,83,93^ still provides a good description of the steady-state temperature profiles predicted in NEMD simulations, as illustrated by the dashed blue line in Figure 4. This observation supports the notion that minor discrepancies between the properties of real and SPC/E model water are unlikely to have any significant effect on the cooling rates experienced by the laser-excited nanoparticles investigated in the next section.

For cases when water in the vicinity of nanoparticles reaches the critical temperature, we also calculate the effective conductance, *h*_*K*_^eff^, by combining the Au–water interfacial conductance with that of the supercritical layer. In the calculation of *h*_*K*_^eff^, the heat flux is defined at the interface between normal and supercritical water, i.e., at the location where *T*_w_ = *T*_c_^SPC/E^, and the temperature jump is defined as Δ*T* ^eff^ = *T*_Au_ – *T*_c_^SPC/E^.

The results of the calculations of *h*_*K*_ and *h*_*K*_^eff^ are shown in Figure 5. Similar to the case of a planar interface, Figure 3a, a gradual decrease of interfacial thermal conductance with an increase in temperature of gold is observed for the 7-nm nanoparticle, Figure 5a. The values of *h*_*K*_ obtained for nanoparticles, however, are consistently higher than the corresponding values predicted for the planar interface. This observation is in agreement with earlier studies where the interfacial thermal conductance is found to increase with increasing curvature in simulations of nanoparticle-fluid Lennard–Jones system^43,44^ and Au nanoparticles in water.^45,46^ The increase in *h*_*K*_, however, does not promote the formation of a supercritical water layer in the vicinity of the nanoparticle. On the contrary, in simulations performed with *T*_w_^HB^ = 300 K, the supercritical water layer does not form up to the maximum temperature of the nanoparticle investigated in the simulations, *T*_Au_ = 1400 K. As a result, the values of *h*_*K*_ stay above 106 MWm^–2^ K^–1^ up to the maximum *T*_Au_, Figure 5a, in a sharp contrast with the results for the planar interface, Figure 3a. When the water heat bath temperature is set to 500 K, the temperature of water adjacent to the nanoparticles does exceed *T*_c_^SPC/E^ for *T*_Au_ ≥ 1000 K, but the thickness of the supercritical layer remains small, below 1 nm up to the maximum temperature of nanoparticles, *T*_Au_ = 1400 K. The decrease in *h*_*K*_ upon the formation of the supercritical water layer in this case is more gradual and moderate as compared to the planar interface (cf. Figures 3a and 5a). The effective conductance *h*_*K*_^eff^ combining the contributions of the Au–water interface and the supercritical layer does exhibit a drop at *T*_Au_ = 1000 K (Figure 5b), albeit to a much higher level as compared to the planar interface (Figure 3b). Similar to the planar interface, when *h*_*K*_ is plotted against the water temperature at the interface, Figure 5c, the results of the two sets of simulations merge into a single monotonous trend. This observation agrees well with previous results that relate the interfacial thermal conductance to the thermodynamic state of the liquid near the interface.^86^

**Figure 5:**
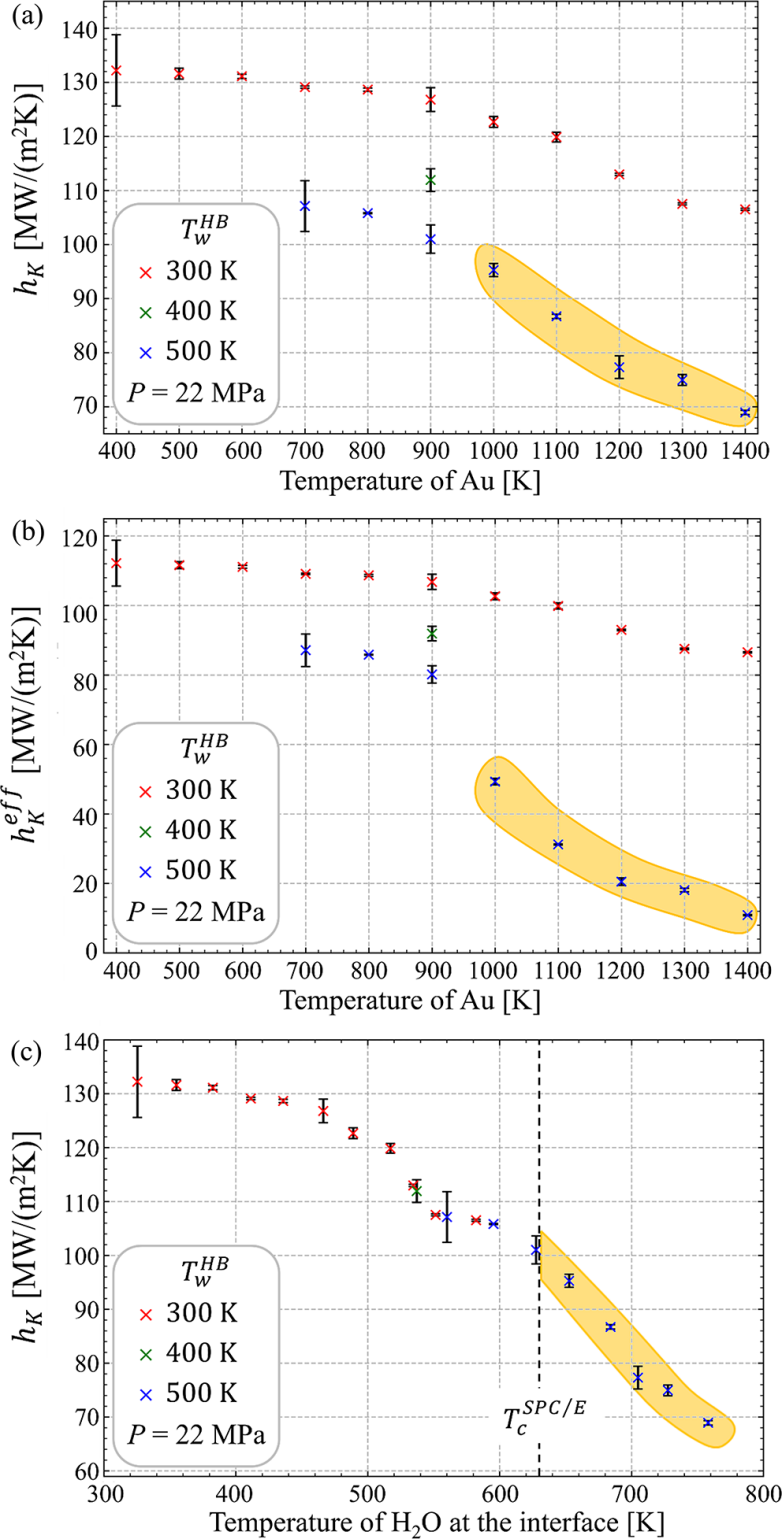
Values of thermal boundary conductance *h*_*K*_ calculated for an interface between water and 7-nm nanoparticles in NEMD simulations performed with a setup shown in Figure 4. The simulations are performed at different temperatures of Au and three temperatures of water heat bath, 300 K (red symbols), 400 K (green symbols), and 500 K (blue symbols). The error bars are calculated as the standard deviation of *h*_*K*_ based on two values of *Q*_r_^int^ evaluated for the Au and water heat bath regions. The values of *h*_*K*_ are plotted as functions of the temperature of Au in (a) and the temperature of water averaged over a 0.08-nm layer adjacent to the Au–water interface in (c). In (b), the effective/combined thermal conductance *h*_*K*_^eff^ of the Au–water interface and a supercritical water layer (if present) is plotted as a function of Au temperature. The yellow areas mark the data points obtained when the formation of a supercritical water layer is observed.

While the conditions of simulations discussed above are described in terms of the temperature of Au nanoparticle *T*_Au_ and the heat bath temperature *T*_w_^HB^, the latter is just a “technical” parameter that controls the steady-state temperature gradient established in the system. It is not possible to directly use *T*_w_^HB^ as a parameter in the continuum-level description of the evolution of temperature around nanoparticles heated by laser irradiation and cooled by heat transfer to the water environment. Different values of *T*_w_^HB^, however, produce different levels of heat flux *Q* (*Q*_r_^int^ for nanoparticles or *Q*_*z*_ for a planar interface) in the steady state, thus enabling analysis of the heat flux dependence of *h*_*K*_. In continuum-level simulations of nonsteady state heat transfer (see Section 3 below), the instantaneous value of *Q* is continuously evaluated and can be used as a parameter defining the value of *h*_*K*_.

The dependence of *h*_*K*_ on the gold temperature and heat flux is shown in Figure 6 based on the results of two series of simulations performed for 7-nm Au nanoparticles at *T*_w_^HB^ of 300 and 500 K, as well as an additional single simulation performed *T*_w_^HB^ = 400 K and *T*_Au_ = 900 K. The results of the simulations are fitted to a multiple linear regression model with *h*_*K*_ defined as a dependent variable, while *Q* and *T*_Au_ are independent variables, i.e.,

1 $$h_{K}\left(T_{\mathrm{Au}},Q\right)=a+\mathrm{bQ}+cT_{\mathrm{Au}}$$

where *a* = 163.5 MWm^–2^ K^–1^, *b* = 0.93 × 10^–3^ K^–1^, and *c* = −0.099 MWm^–2^ K^–2^ are the parameters obtained for the 7-nm nanoparticle. The quality of the fit can be seen from the deviations between the values predicted by eq 1 and shown in Figure 6a by silver crosses from the actual data points predicted in the simulations and shown by open circles. The approximation of data provided by eq 1 describes the variation of *h*_*K*_ over continuous ranges of *Q* and *T*_Au_, Figure 6b, which is needed for the parametrization of the continuum-level description of the transient heat transfer discussed in Section 3. We note that the simple linear dependence provided by eq 1 cannot be extrapolated far from the range of parameters used for fitting this relationship. In particular, since both the left- and right-hand parts of eq 1 contain a linear dependence on *Q* (*h*_*K*_ = *Q*/Δ*T*), the flux diverges when Δ*T* = *T*_Au_ −*T*_w_^int^ → *b* ^–1^ = 1075 K.

**Figure 6:**
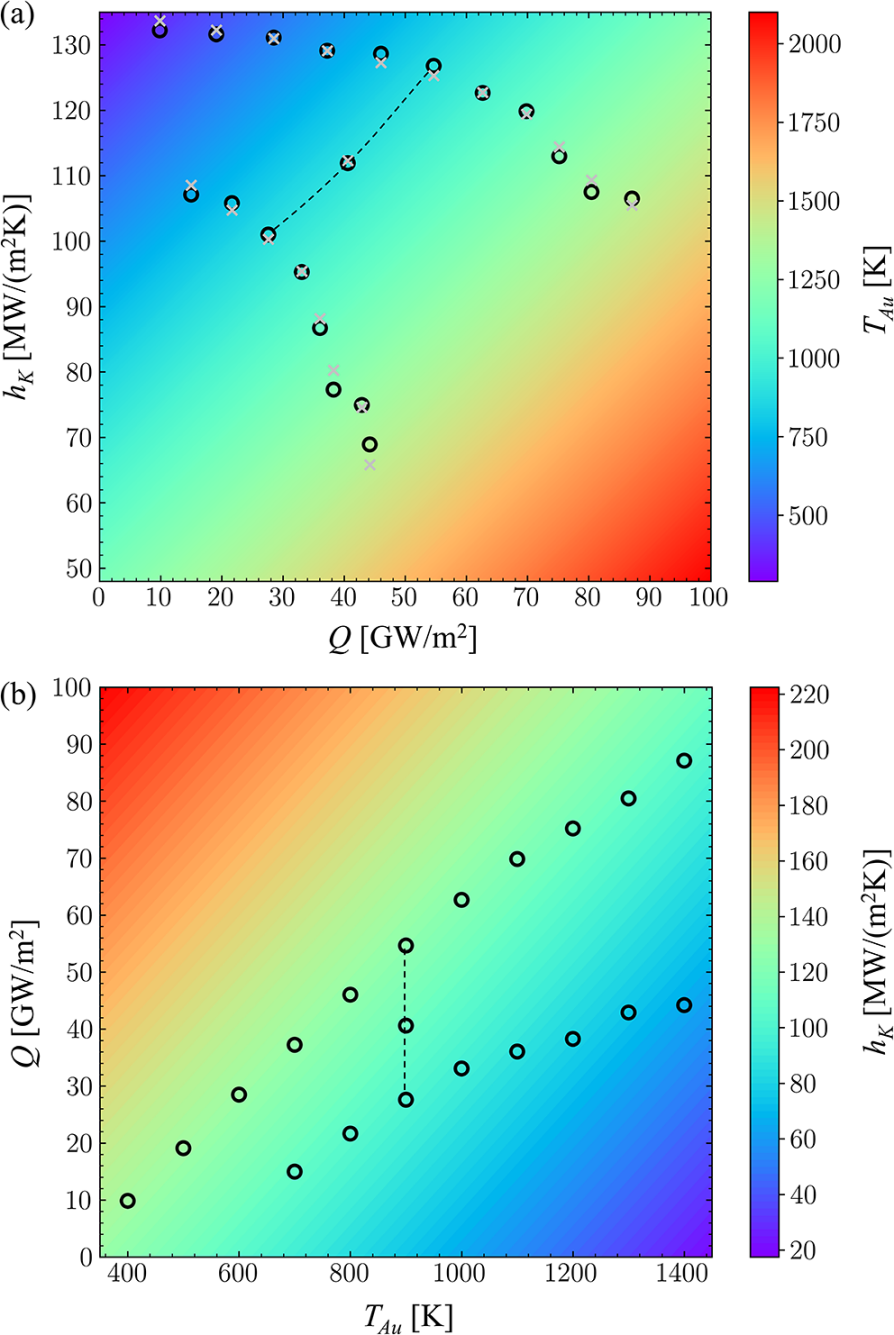
Interfacial thermal conductance *h*_*K*_ as a function of heat flux *Q*_r_^int^ across the interface (a) and the heat flux *Q*_r_^int^ as a function of gold temperature *T*_Au_ (b) calculated for 7-nm Au nanoparticles. The color coding shows the distribution of *T*_Au_ in (a) and *h*_*K*_ in (b) predicted with multiple linear regression fitting of the data points generated in simulations performed at *T*_w_^HB^ = 300 K (upper branches of data points), *T*_w_^HB^ = 500 K (lower branches of data points), and *T*_w_^HB^ = 400 K (a point between the two branches). Silver crosses in (a) show the *h*_*K*_ values predicted by eq 1. The dashed line connects three data points obtained for *T*_Au_ = 900 K.

On the heat flux dependence of the thermal boundary conductance across the interface, we also note that a clear positive correlation of *h*_*K*_ with *Q* is observed at fixed values of *T*_Au_, e.g., along the dashed line in Figure 6a for *T*_Au_ = 900 K. When comparing this observation with earlier computational studies,^42,46,51^ we note that the increase in the heat flux is usually associated with the simultaneous increase in the temperature of Au. As a result, the competition of the negative correlation with temperature (Figure 5a) and positive correlation with heat flux magnitude (Figure 6a) can yield an increasing,^46,51^ decreasing,^42^ or neutral^46,51^ *h*_*K*_(*Q*) dependences.

To evaluate the dependence of the interfacial thermal conductance on the curvature of the interface, the results reported above for the planar interfaces and nanoparticles with a diameter of 7 nm are complemented by a set of NEMD simulations performed for 20-nm nanoparticles. The simulations are done with the same computational setup as that discussed above for 7-nm nanoparticles, the heat bath temperature *T*_w_^HB^ is set to 500 K, and the Au temperature *T*_Au_ is maintained at 800, 1000, and 1200 K. The values of *h*_*K*_ calculated in this series of simulations fall between the values obtained for 7-nm nanoparticles and planar interfaces, as shown in Figure 7a.

**Figure 7:**
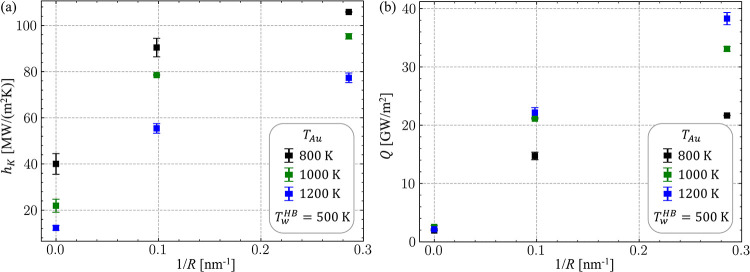
Interface curvature dependence of the interfacial thermal conductance *h*_*K*_ (a) and steady-state heat flux *Q* (b) predicted in NEMD simulations of a planar Au–water interface and Au nanoparticles with diameters of 7 and 20 nm. The simulations are performed with the same water heat bath temperature, *T*_w_^HB^ = 500 K.

The observed general trend of thermal conductance increasing with curvature of the interface agrees with earlier NEMD results reported for Lennard–Jones^43,44,48^ and Au–water^45,46^ systems, as well as the results of simulations of melting and resolidification of ZnO nanoparticles irradiated by a picosecond laser pulse in liquid tetradecane.^18^ Several mechanisms responsible for the increase in thermal conductance with decreasing size of the nanoparticles has been discussed, including an enhanced adsorption of fluid molecules,^44–46^ stronger interfacial bonding,^43^ and greater contribution of undercoordinated atoms^45,48^ on the surface of smaller nanoparticles, as well as size-dependent modification of nanoparticle vibrational properties leading to an improved vibrational coupling between smaller nanoparticles and molecular fluid.^45^ We note that exceptions from this trend have also been reported, including the results for 1–4 nm Au nanoparticles solvated in hexane,^47^ where *h*_*K*_ is observed to decrease with decreasing size of spherical nanoparticles and the role of undercoordinated surface atoms in thermal conductance is highlighted, as well as the results for small 1–2.5 nm thiolate-capped Au nanoparticles in hexane,^94^ where the thermal conductance is found to be strongly affected by the ligand chain length and flexibility, and no systematic size dependence is observed.

In the present study, the increase in *h*_*K*_ with increasing curvature of the interface is observed even when the temperature of Au is above the critical temperature of water, Figure 7a. In the case of a planar interface, the thermal conductance is strongly reduced due to the formation of a low-density supercritical layer, as discussed in Section 2.2. For nanoparticles, however, the formation of the low-density layer is suppressed, as can be seen from the snapshots of slices of atomic configurations in the interfacial regions shown in Figure 8. All slices have the same thickness of 10 nm, making the difference in the density of water between the planar and curved interfaces visually apparent. For the same temperature of Au, *T*_*Au*_ = 1000 K, a low-density region with a thickness of several nanometers can be clearly seen in the snapshot shown in Figure 8a, as well as in the density profile in Figure 9b. There is still a layer of molecules adsorbed on the gold surface and the corresponding density peak within the first nanometer from the Au surface, but the contribution of this layer to the interfacial heat transfer is limited by the low-density region farther away from the interface. In the case of both 7 and 20-nm nanoparticles, there is still a minimum in the water density profile in the close vicinity of the interface, but the minimum density is more than three times higher for 7-nm nanoparticles as compared to the planar interface, Figure 9b,d. The density peak that corresponds to the molecules adsorbed on the interface is also more than twice as high for the nanoparticles, suggesting that the enhanced interfacial adsorption discussed as a possible reason for the curvature dependence of the thermal conductance at moderate temperatures^44–46^ may also play a role at higher temperatures considered in the present study.

**Figure 8:**
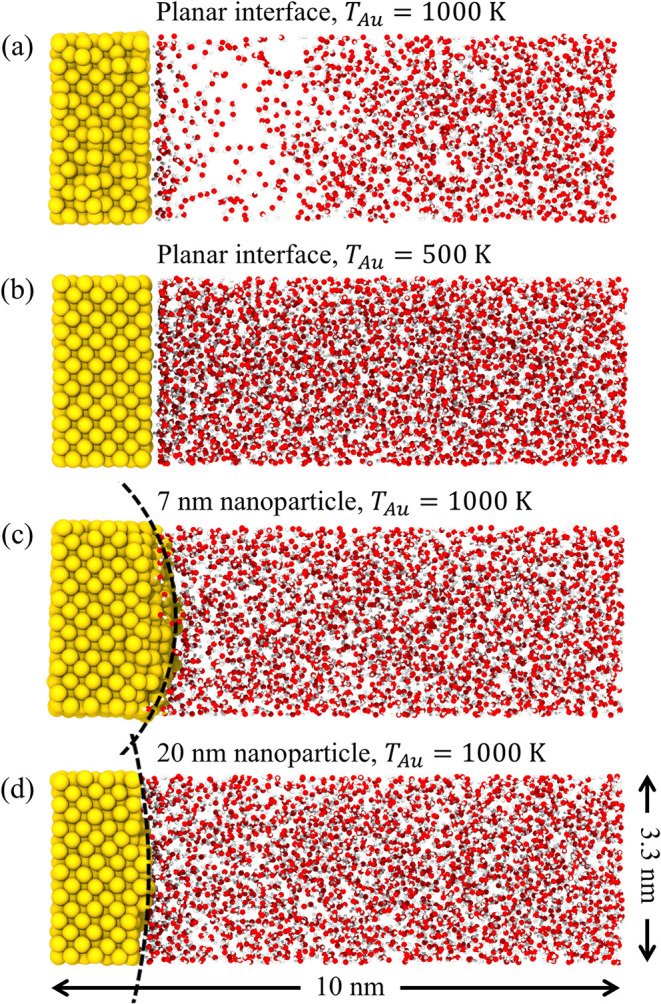
Snapshots of slices of atomic configurations generated in NEMD simulations of steady state heat transfer from Au to water shown for (a) planar interface at *T*_Au_ = 1000 K, (b) planar interface at *T*_Au_ = 500 K, (c) 7-nm nanoparticle at *T*_Au_ = 1000 K, and (d) 20-nm nanoparticle at *T*_Au_ = 1000 K. All simulations are performed at a mean water pressure of 22 MPa and water heat bath temperature of *T*_w_^HB^ = 500 K. Each slice has the same dimensions of 3.3 nm × 3.3 nm × 10 nm. The dashed curves in (c, d) show the curvature of the interfaces.

**Figure 9:**
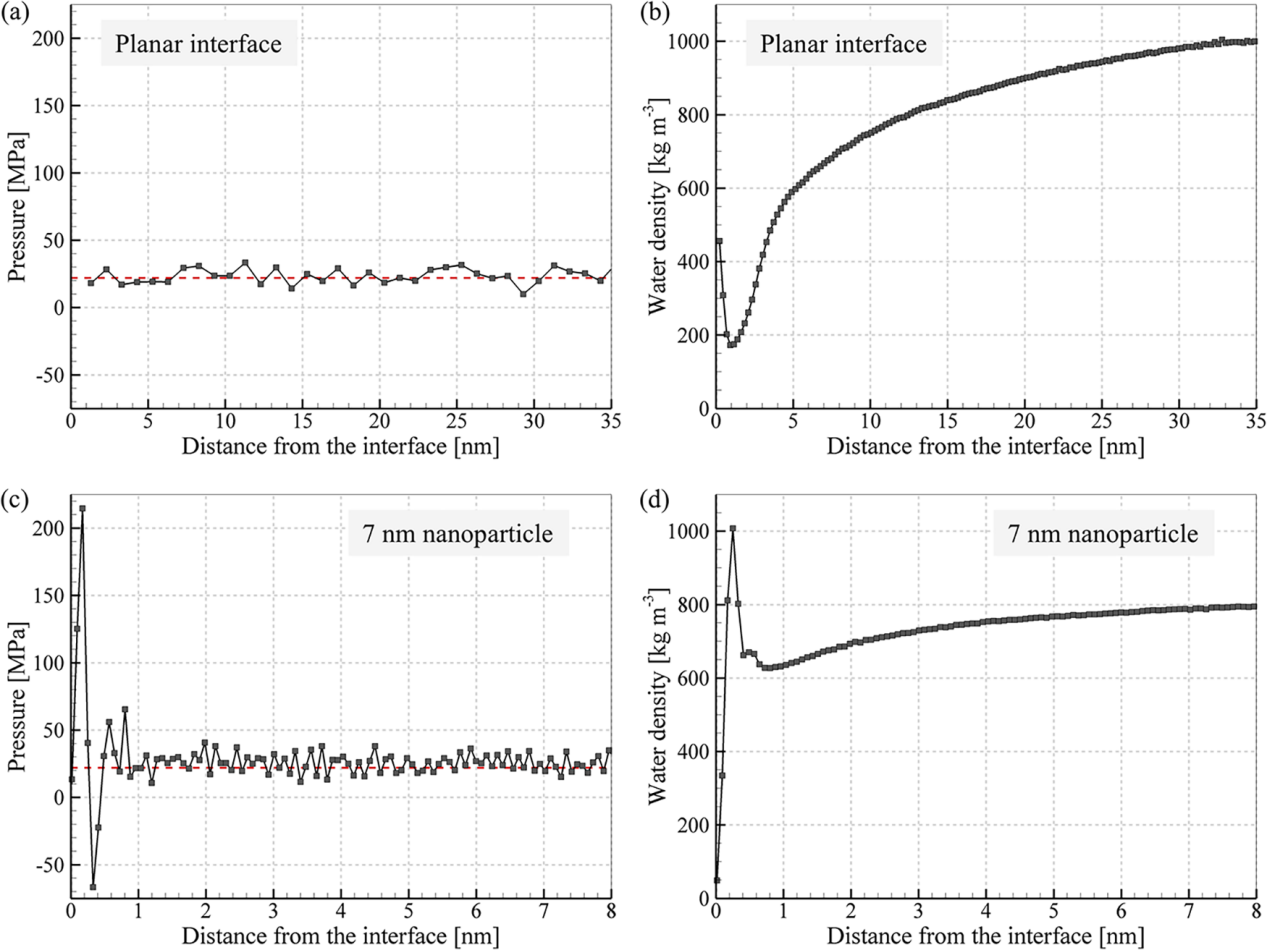
Distribution of hydrostatic pressure and water density near a planar interface with Au (a, b) and an interface with 7-nm Au nanoparticle (c, d) predicted in NEMD simulations performed at *T*_Au_ = 1000 K. The water heat bath temperature *T*_w_^HB^ is maintained at 300 K in the simulation of the planar interface and at 500 K in the simulation of the nanoparticle. The red dashed lines mark the average water pressure of 22 MPa maintained in the simulations. The plots for the planar interface show the result of averaging over data obtained for two interfaces present in each simulation on the two sides of the Au layer, Figure 2.

The major factor in sustaining the high interfacial thermal conductance at high temperatures in the case of the nanoparticles, however, is suppression of the formation of the low-density region. This effect can be attributed to the compression produced by the Laplace pressure related to the tension of the curved interface between supercritical and normal water. While the surface tension of water vanishes at *T* = *T*_c_^SPC/E^, the large temperature gradient around the nanoparticle (e.g., Figure 4) produces a nanoscale diffuse interface where the temperature of water drops down to the normal water conditions. The tension produced by the diffuse interface can be seen as a negative (tensile) component of the pressure peak present in the vicinity of the nanoparticle, Figure 9c. The location of this peak of tensile stresses marks the turn in the density profile in Figure 9d from the gradual decrease reflecting the thermal expansion of normal water (*T* < *T*_c_^SPC/E^) to the sharp rise in density in the immediate vicinity of the nanoparticle. The direct mapping of theoretical values of Laplace pressure produced by curved thermally equilibrated liquid–vapor interfaces to the simulated system featuring a very steep temperature variation (from *T* > *T*_c_^SPC/E^ at the interface down to 500 or 300 K just 8 nm from the interface) cannot be expected to yield a good quantitative agreement. Nevertheless, we note that the values of Laplace pressure, *P*_*L*_ = 2γ/*R*, evaluated for *R* = 3.5 nm and γ predicted with SPC/E potential^95^ are ranging from 36 MPa at 300 K (γ = 63.6 mJ/m^2^) to 8 MPa at 550 K (γ = 13.8 mJ/m^2^), which is of the same order of magnitude as the pressure variation observed in the vicinity of the nanoparticle in Figure 9c. In contrast, no significant pressure variation is observed for the planar interface, where the pressure remains close to the average level imposed in the simulation at all distances from the interface, Figure 9a, even though the water density undergoes large variations, Figure 9b.

The suppression of the formation of low-density regions around hot nanoparticles has also been observed in simulations of Lennard–Jones^41,42^ and Au–octane systems,^42^ where it is also attributed to the curvature-induced compression of fluid around the nanoparticle. Note that transient formation of cavitation bubbles has been observed for small (1 to 3 nm) ZnO nanoparticles in liquid tetradecane^18^ and larger (20 nm) Au nanoparticles in water^20,21^ subjected to impulsive laser-induced heating leading to nanoparticle melting and recrystallization^18^ or fragmentation.^20,21^ The Laplace pressure in these cases is substantially higher than the internal pressure in the transient cavitation bubbles, leading to the collapse of the cavitation bubble within 1 ns for ZnO in tetradecane^18^ and on a time scale ranging from 3.5 to 11 ns depending on the energy input for the fragmentation Au nanoparticles in water.^20,21^ Similarly to the negative spike in the pressure profile in Figure 9c, the location of the surface of the cavitation bubble is signified by the localized tension in the pressure plots obtained in the simulations of Au nanoparticle fragmentation.

To incorporate the dependence on curvature into the description of *h*_*K*_, we rewrite eq 1 formulated based on the results for 7-nm nanoparticle (*R* = 3.5 nm) in a general form

2 $$h_{K}\left(T_{\mathrm{Au}},Q,R\right)=a\left(R\right)+b\left(R\right)Q+c\left(R\right)T_{\mathrm{Au}}$$

where coefficients *a*(*R*), *b*(*R*), and *c*(*R*) are assumed to be linear functions of curvature, i.e., *a*(*R*) = *a*_0_ *+ a*_1_/*R*, *b*(*R*) = *b*_0_ *+ b*_1_/*R*, and *c*(*R*) = *c*_0_ *+ c*_1_/*R*. Fitting this equation with data obtained for 7 and 20-nm nanoparticles as well as the planar interface yields the following coefficients: *a*_0_ = 141.3 MWm^–2^ K^–1^, *b*_0_ = 2.27 K^–1^, *c*_0_ = −0.11 MWm^–2^ K^–2^, *a*_1_ *=* 7.95 × 10^–8^ MWm^–1^ K^–1^, *b*_1_ = −4.63 × 10^–9^ m K^–1^, *c*_1_ = 3.48 × 10^–11^ MWm^–1^ K^–2^. The quality of the fit is evaluated using the coefficient of determination, *R* ^2^ = 0.95, estimated as the average across 5-fold cross-validation. For the 7-nm nanoparticle, the coefficients obtained from this fit are within 2% of those determined by fitting to the data for this nanoparticle alone and listed above, below eq 1. For the 20-nm nanoparticle (*R* = 10 nm), the following coefficients are obtained: *a* = 149.25 MWm^–2^ K^–1^, *b* = 1.807 × 10^–3^ K^–1^, and *c* = −0.106 MWm^–2^ K^–2^.

Note that a simple linear dependence on curvature suggested in refs 44–46 , *h*_*K*_(1/*R*) = *h*_*K*_(0) + δ/*R*, is not sufficient, as both parameters of this equation depend on *T*_Au_ and *Q*. Maintaining constant values of both *T*_Au_ and *Q* while varying the nanoparticle size is not feasible. For example, in NEMD simulations of steady-state heat transfer performed at fixed *T*_Au_ and *T*_w_^HB^, the value of *Q* changes substantially with the curvature of the interface (Figure 7b). Moreover, under conditions of heating and cooling of nanoparticles considered in Section 3, both *T*_Au_ and *Q* evolve during the simulations. To account for this evolution, the dependence of *h*_*K*_ on *T*_Au_ and *Q* must be defined for each nanoparticle size of interest. The linear dependences given by eqs 1 and 2 provide the simplest approximation that still describes the results of NEMD simulations reasonably well, as illustrated by Figure 6a.

Yet another parameter that may affect the interfacial thermal conductance is the pressure in the liquid environment. Under conditions of rapid laser heating of nanoparticles, the pressure distribution in the surrounding liquid can undergo rapid changes due to thermal expansion and heat transfer from the nanoparticle. In particular, the maximum pressure predicted in continuum-level simulations of short pulse laser irradiation of Au nanoparticles below the threshold for nanoparticle melting and fragmentation^56,96^ can exceed 50 MPa immediately after the laser excitation, and then decays on the time scale of tens of picoseconds. The pressure pulses can be substantially stronger at higher fluences, in the regime of nanoparticle melting and fragmentation.^20,21^

To evaluate the effect of pressure on the interfacial thermal conductance, a series of NEMD simulations is performed for 7-nm nanoparticles at varying levels of average water pressure. The simulations are carried out at five values of *T*_Au_, ranging from 1000 to 1400 K, while maintaining the same water heat bath temperature, *T*_w_^HB^ = 500 K. The results of the calculations, plotted in Figure 10, suggest a relatively weak sensitivity of *h*_*K*_ to the variation of pressure. In particular, at *T*_Au_ = 1000 K, the value of *h*_*K*_ changes by about 13%, from 86.6 MWm^–2^ K^–1^ to 98.1 MWm^–2^ K^–1^, when the pressure is varied from 2.5 to 39.6 MPa. This relatively small change in *h*_*K*_ upon variation of pressure by more than an order of magnitude can be attributed to the major contribution of the curvature-induced compression of water in the vicinity of the interface, discussed above and illustrated in Figure 9c,d. Indeed, a more significant change in *h*_*K*_ is observed at *T*_Au_ = 1300 K, when the decrease in the average pressure from 41.1 to 5.2 MPa reduces *h*_*K*_ by more than 25%. This reduction is accompanied by a reduction in water density near the interface, which may be related to the decrease in surface tension with temperature^95^ allowing for an easier expansion of the supercritical water layer. As discussed above, however, quantitative mapping of the temperature dependence of surface tension to the conditions of extreme temperature gradients created around the nanoparticles should be done with caution.

**Figure 10:**
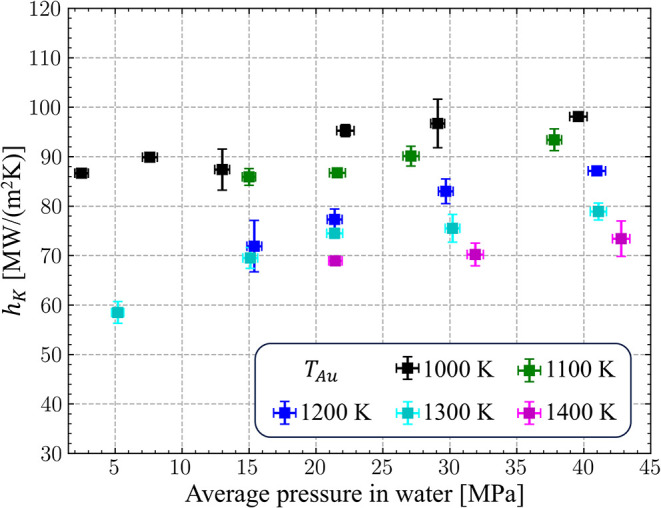
Values of thermal boundary conductance *h*_*K*_ calculated for 7-nm Au nanoparticles in water at different levels of average hydrostatic pressure in water. All simulations are performed at *T*_w_^HB^ = 500 K. The error bars for *h*_*K*_ are calculated as discussed in the caption of Figure 5. The error bars for pressure are standard deviations from time-averaged values calculated over the last 30,000 timesteps (30 ps) of the simulations.

For a planar Au–water interface, the value of *h*_*K*_ has been reported to be insensitive to variation of pressure from 40 to 500 MPa under conditions significantly below the critical temperature.^97^ On the other hand, a sharp increase in *h*_*K*_ upon compression is observed in NEMD simulations of graphene–water system where water is heated above the critical point.^98^ Overall, since the focus of the present study is on nanoparticles, where the variation of pressure in the range typical for laser-induced heating and cooling is expected to have a relatively small effect on the thermal conductance, we do not include the effect of pressure in the parametrization of the continuum-level model discussed in the next section. We note, however, that generation of cavitation bubbles predicted for sufficiently high energy inputs^18,20,33,35–40,59^ can interrupt the heat transfer and have a substantial effect on the thermal history of the nanoparticles.

## 3 Laser-Induced Heating, Melting, Cooling, and Resolidification of Au Nanoparticles

The information on the thermal boundary conductance *h*_*K*_(*T*_Au_ *,Q,R*) and thermal conductivity of water *k*(*T*) obtained in NEMD simulations is used here to design a continuum-level model for simulation of nanoparticle heating, melting, cooling, and resolidification. The formulation of the model is provided first in Section 3.1, followed by verification of the model by direct comparison with atomistic simulations of laser interactions with Au nanoparticles in water discussed in Section 3.2. Finally, in Section 3.3, a hybrid atomistic-continuum model with “implicit” representation of the water environment is formulated and applied for a computationally efficient analysis of laser-induced structural modification of nanoparticles.

### 3.1 Continuum Model for Laser Interactions with Au Nanoparticles in Water

The continuum description of laser interaction with a Au nanoparticle is based on the two-temperature model (TTM),^99^ commonly used in the analysis of short pulse laser interactions with metals.^100^ In TTM, the time evolution of the lattice^101^ and electron temperatures, *T*_l_ and *T*_e_, is described by two coupled differential equations, formulated here under assumption of uniform distribution of *T*_e_ and *T*_l_ within the nanoparticle. The assumption of constant *T*_e_ is well justified by the small size of the nanoparticles considered in the present study and the large electron thermal conductivity of Au, leading to the establishment of uniform distribution of electron temperature on a picosecond time scale. The assumption of constant *T*_l_, while simplifying the analysis, may not accurately reflect the complexities of real heat transfer from a larger 20-nm nanoparticle to the water environment. Despite the uniformity of the electron temperature and a negligible contribution of phonons to the heat transport in Au,^102,103^ the vibrational heat transfer from the nanoparticle to the water environment may produce a substantial lattice temperature variation within the nanoparticle. This effect is discussed in Section 3.2.2, where an *ad hoc* correction to the continuum model accounting for the lattice temperature nonuniformity is suggested.

With the assumptions discussed above, the TTM equations for a nanoparticle in water can be formulated in a simple form of

3 $$c_{e}^{Au}\left(T_{e}\right)\frac{\partial T_{e}\left(t\right)}{\partial t}=-G\left(T_{e}\right)\left(T_{e}-T_{l}\right)+S_{L}\left(t\right)$$

4 $$c_{l}^{\mathrm{Au}}\left(T_{l}\right)\frac{\partial T_{l}\left(t\right)}{\partial t}=G\left(T_{e}\right)\left(T_{e}-T_{l}\right)-S_{m}\left(T_{l},f_{l}\right)-\frac{3Q_{r}^{\mathrm{int}}}{R}$$

where *c*_e_^Au^ and *c*_l_^Au^ are the volumetric electron and lattice heat capacities, *G* is the electron–phonon coupling factor, *Q*_r_^int^ is the heat flux at the nanoparticle–water interface, *R* is the nanoparticle radius, and the source terms correspond to the laser energy deposition, *S*_L_, and the latent heat of melting/solidification, *S*_m_. The electron temperature dependence of *c*_e_^Au^(*T*_e_) and *G*(*T*_e_) are taken from ref 104, the lattice temperature dependence for solid Au is from ref 63, and a constant value of vibrational heat capacity of molten Au, *c*_l_^Au,l^ = 32.97 J mol^–1^ K^–1^ (ref 63) is assumed in the TTM calculations.

The laser energy deposition is described by the source term *S*_L_(*t*) added to the TTM equation for the electronic temperature to represent the excitation of the conduction band electrons by a laser pulse. In the present study, the laser pulse is assumed to have a Gaussian temporal profile^105^ with the full width at half-maximum equal to the laser pulse duration of τ_L_ = 10 ps. To ensure complete laser energy deposition, the laser pulse is shifted forward in time by 2.5 × τ_L_ with respect to the start of the simulation.

The melting and solidification processes are included through the term *S*_m_(*T*_l_, *f*_l_) added to the equation for the lattice temperature. This term is activated as soon as the lattice temperature reaches the equilibrium melting temperature of Au, *T*_m_ (or *T*_m_^EAM^ when the simulation is intended to describe the results of atomistic modeling). Upon heating, any additional energy transferred to the lattice from the hot electrons goes into changing the fraction of the liquid phase in the nanoparticle, *f*_l_, until the nanoparticle is fully melted, i.e., *f*_l_ = 1. Upon cooling, the heat transferred to the surrounding water is assumed to be fully compensated by the release of the heat of melting until complete solidification of the nanoparticle, i.e., *f*_l_ = 0. The conversion of the heat added/removed to the change in *f*_l_ is done with the bulk values of the heat of melting, Δ*H*_m_ or Δ*H*_m_^EAM^. This simple representation of melting and solidification does not allow for superheating/undercooling above/below *T*_m_, which can be substantial under conditions of rapid heating and cooling triggered by short pulse laser irradiation. Indeed, as discussed in Section 3.2, the deviations between the predictions of the continuum model and atomistic simulations are largely related to the highly nonequilibrium conditions of the phase transformations. While it is possible to include more realistic descriptions of melting and solidification under conditions of superheating and undercooling,^106,107^ the effects of the surface and high curvature of liquid–solid interface on the kinetics of nonequilibrium phase transformations are poorly explored and present challenge for reliable model parametrization. Therefore, in Section 3.3, we design a hybrid model retaining an atomistic representation of the phase transformations in the nanoparticles and combining it with a continuum description of the water environment.

The temperature evolution in water surrounding the nanoparticle is represented by the heat diffusion equation formulated in spherical coordinates as

5 $$c_{P}^{w}\left(T\right)\frac{\partial T_{w}\left(r,t\right)}{\partial t}=\frac{1}{r^{2}}\frac{\partial }{\partial r}\left(r^{2}k\left(T\right)\frac{\partial T_{w}\left(r,t\right)}{\partial r}\right)$$

where *k*(*T*) is the thermal conductivity of SPC/E water plotted in Figure 1 and *c*_P_^w^(*T*) is the volumetric heat capacity of water at constant pressure. The values of heat capacity for the SPC/E water model are calculated for a pressure of interest (*P* = 22.065 MPa) based on the enthalpy curve, *H*_w_(*T*_w_), obtained in a series of constant-pressure constant-temperature (NPT) MD simulations performed for *T*_*w*_ increasing from 298 to 700 K with 2 K increments. At each temperature, the system is allowed to equilibrate for 20 ps. The resulting *H*_w_(*T*_w_) dependence is approximated by a cubic univariant spline to reduce the noise and is numerically differentiated to obtain the heat capacity as *c*_P_^w^(*T*) = (∂*H*_w_(*T*_w_)/∂*T*_w_)_*P*_.

The coupling of eq 5 for the heat diffusion in water to the TTM equations for nanoparticle, eqs 3 and 4, is implemented through boundary condition defined at the Au–water interface, *T*_w_(*R*) = *T*_Au_ – *Q*_r_^int^/*h*_*K*_(*T*_Au_,*Q*_r_^int^), where *T*_Au_ is equal to the instantaneous lattice temperature of the nanoparticle, *T*_l_, predicted by eq 4. We note that neglecting the interfacial thermal resistance^108^ would not be appropriate for systems investigated in the present study. Indeed, the nanoparticle size range where the interfacial thermal conductance plays a significant or even dominant role in cooling a nanoparticle heated by a laser pulse can be estimated by comparing the characteristic time scales of the diffusional heat dissipation in water,^25^ τ_w_ = *R* ^2^(*c*_p_^Au^)^2^/9*c*_p_^w^ *k* and the cooling time defined by the interfacial conductance alone, τ_*h*_*K*__ = *Rc*_p_^Au^/3*h*_*K*_. The relative contributions of thermal conduction in water and thermal boundary conductance can be characterized by the dimensionless parameter α = τ_w_/τ_*h*_*K*__ = *Rc*_p_^Au^ *h*_*K*_/3*c*_p_^w^ *k*. Using constant values of *c*_p_^Au^ = 2.5 MJ m^–3^ K^–1^, *c*_p_^w^ = 4.1 MJ m^–3^ K^–1^, *h*_*K*_ = 120 MW m^–2^ K^–1^, and *k* = 0.6 Wm^–1^ K^–1^ for a rough estimate, we get α ≈ 0.4 for *R* = 10 nm and α ≈ 0.14 for *R* = 3.5 nm, indicating that neither the heat diffusion in liquid nor the interfacial thermal resistance can be neglected when describing cooling of 7 and 20-nm nanoparticles. The condition of α ≈ 1 (i.e., τ_*h*_*K*__ ≈ τ_w_ ≈ 171 ps) is satisfied for *R* ≈ 25 nm, suggesting that cooling of the nanoparticles considered in this study is largely controlled by the interfacial conductance.

At the outer boundary of the computational domain, the adiabatic boundary condition is applied. In simulations discussed in Section 3.2, this boundary is placed at a distance of 8 nm from the nanoparticle–water interface to match the location of the boundary in the atomistic NEMD simulations. In simulations reported in Section 3.3, the boundary is placed at a distance of 100 nm, which is sufficient for ensuring that the temperature increase at the boundary by the end of the simulation is negligible. The initial temperature of the nanoparticle and water is set to 300 K.

### 3.2 Atomistic Modeling of Laser Interactions with Au Nanoparticles in Water

The ability of the continuum model described in the previous section to provide an adequate description of steady-state temperature profiles predicted in NEMD simulations has been demonstrated in Figure 4, where the temperature profiles predicted for the steady state conditions (i.e., by equating the left-hand side of eq 5 to zero and applying boundary conditions mimicking those used in the NEMD simulations) are shown to agree well with the data points predicted in the atomistic simulations. In this section, the model is verified under rapidly evolving conditions of laser-induced heating and cooling of nanoparticles.

The atomistic simulations of laser–nanoparticle interactions are performed with a model combining TTM with MD in a manner similar to that described in ref 105. The MD substitutes the TTM equations for the lattice temperature, eq 4, and the energy transferred from/to the electronic subsystem is deposited/removed to/from the energy of atomic motions with heat-exchange algorithm^109^ implemented in LAMMPS. Due to the small size of the 7-nm nanoparticle, its temperature is described by a single value of *T*_Au_ calculated from the kinetic energy averaged over the whole nanoparticle. This value then enters as *T*_l_ in the TTM equation for the electron temperature, eq 3. For a 20-nm nanoparticle, to account for the possibility of the formation of a radial temperature gradient within the particle due to the rapid heat exchange with the surrounding water, we divide it into nine regions (a central spherical core region and eight concentric layers) of approximately equal volume. The lattice temperature for the TTM equation is then calculated for each region individually and couples to the single pool of the electron energy described by eq 3. As a result, the heat exchange between the concentric layers of the nanoparticle is largely controlled by electron–phonon coupling.

To simulate a nonreflecting propagation of a spherical pressure wave generated by a rapid laser heating, an additional radial force, *F*_r_, is applied to water molecules located within the 1-nm-thick outer shell of the spherical computational system.^110,111^ The magnitude of the extra force is proportional to the average radial velocity of atoms in the shell, *v*_r_, and acoustic impedance *Z*, i.e., *F*_r_ = −*ZS*_a_ *v*_r_, where *S*_a_ is the surface area per boundary molecule. The value of the specific acoustic impedance, *Z* = 1.6 MPa·s·m^–1^, is obtained from a set of MD simulations of acoustic wave propagation in SPC/E water. This value is slightly higher than an experimental value of about 1.5 MPa·s·m^–1^ due to the finite amplitude of the acoustic waves generated in the simulations. A finite radius of the spherical computational system creates an artificial Laplace pressure, which is not present in a bulk colloidal solution. To compensate for this pressure, we apply an extra radial force of *F*_L_ = 8πγ*R*_w_/*N*_w_^at^ to each atom of water molecules in the system, where *R*_*w*_ is the outer radius of the water shell and *N*_w_^at^ is the total number of oxygen and hydrogen atoms in the system.

#### 3.2.1 Laser Interaction with 7-nm Au Nanoparticle

The results of TTM-MD simulations of laser-induced heating, melting, cooling, and solidification of 7-nm Au nanoparticles are illustrated in Figures 11 and 12 for three values of energy density deposited by the laser pulse.

**Figure 11:**
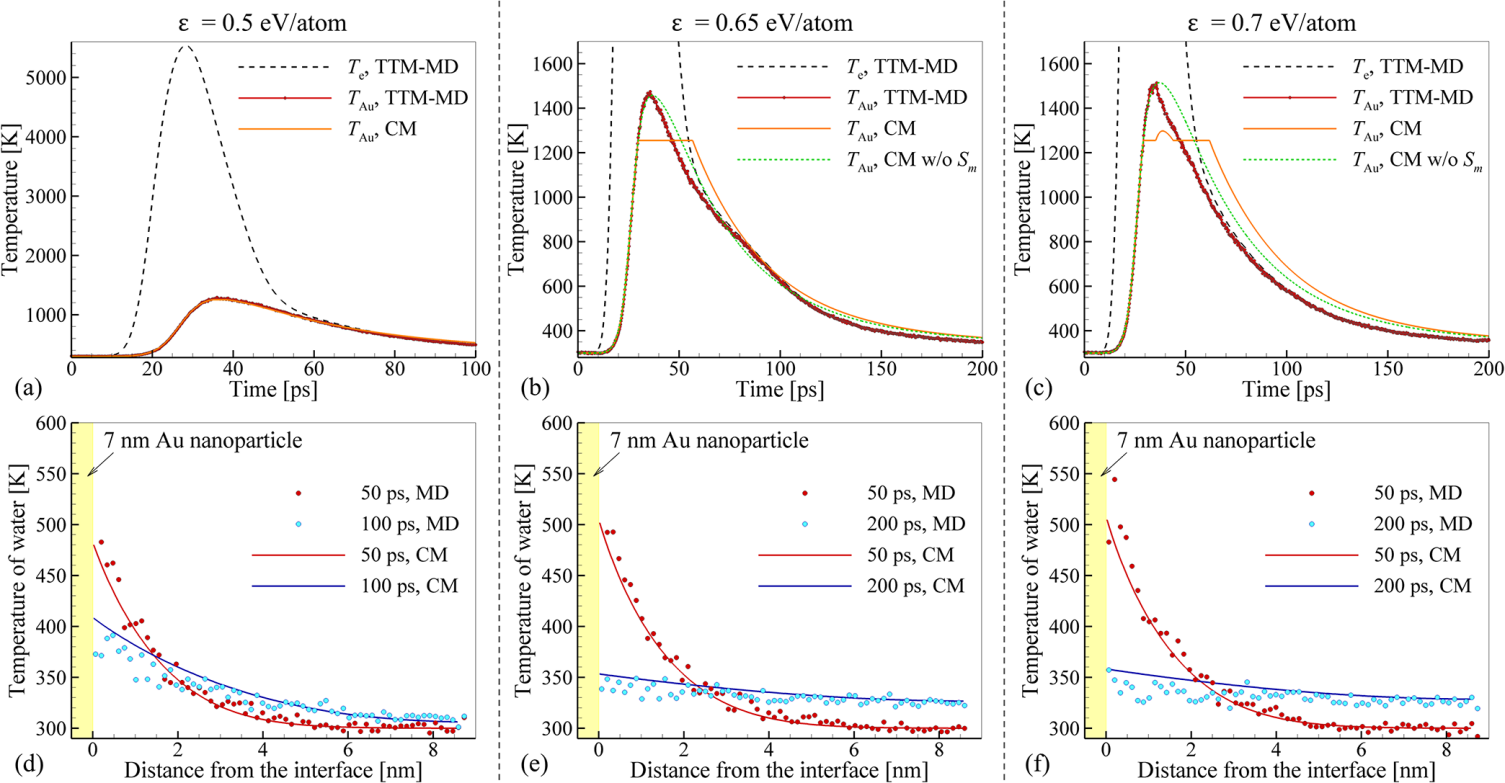
Evolution of electron and lattice temperatures of 7-nm Au nanoparticle (a–c) and temperature of the surrounding water (d–f) after irradiation of the nanoparticle in water by a 10 ps laser pulse at three absorbed energy densities ε: 0.5 eV/atom (a, d), 0.65 eV/atom (b, e), and 0.7 eV/atom (c, f). Results of TTM-MD simulations are compared with predictions of a continuum model (CM) based on the numerical integration of eqs 3–5. The CM solution neglecting the energy transformations due to melting and solidification, i.e., with the term *S*_m_(*T*_l_, *f*_l_) omitted in eq 4, is shown in (b, c) by green dashed curves. The temperature profiles in water are shown for 50 and 100 ps in (d) and for 50 and 200 ps in (e, f). The laser pulse peak intensity occurs at 2.5τ_L_ = 25 ps.

**Figure 12:**
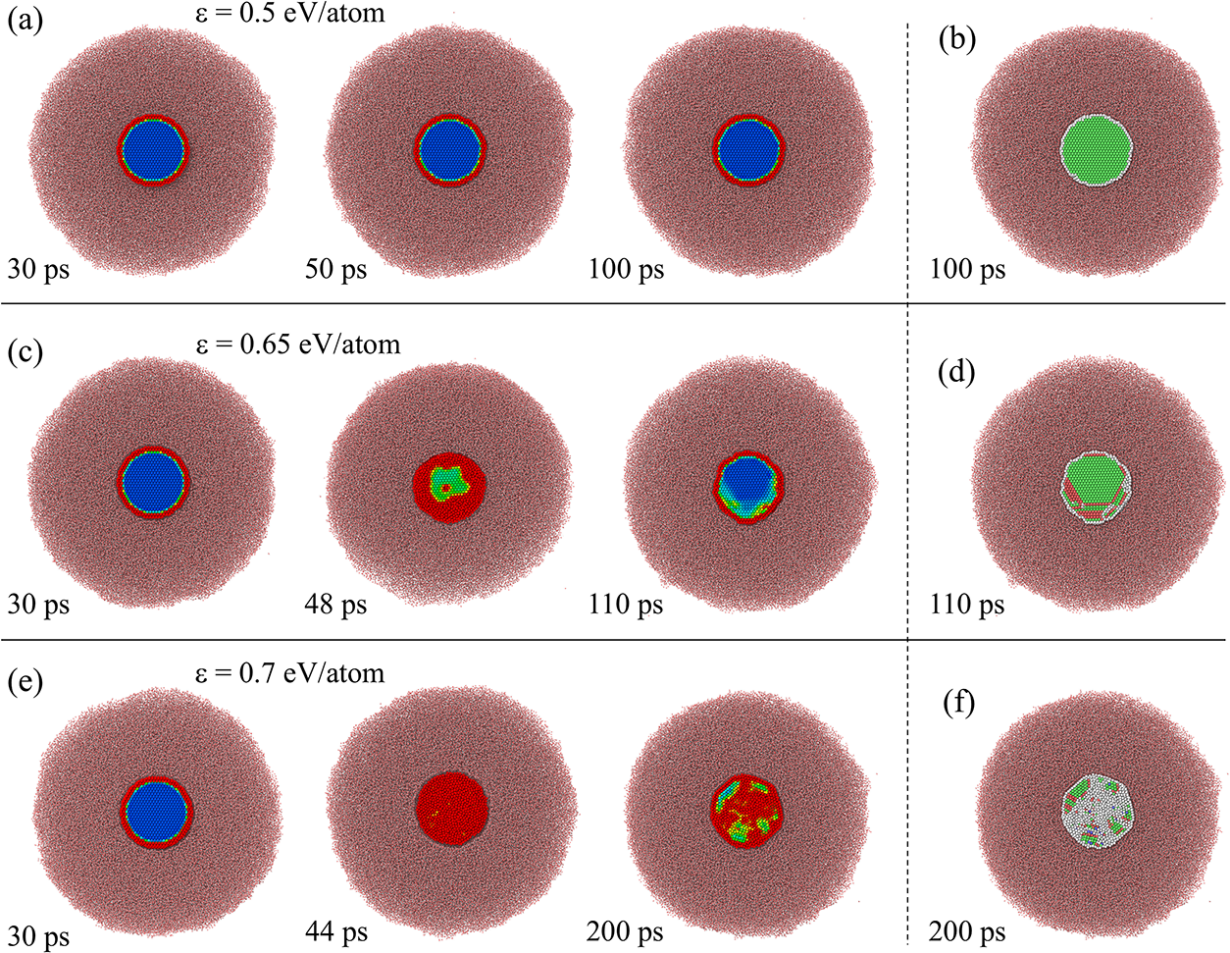
Cross sections of atomic configurations predicted in TTM-MD simulations of 7-nm Au nanoparticles irradiated in water by 10 ps laser pulses at absorbed energy densities of 0.5 (a, b), 0.65 (c, d), and 0.7 eV/atom (e, f). The Au nanoparticles are quenched to 0 K prior to making the plots and are colored by their potential energy averaged over neighbors within a 3.5 Å radius, ranging from −3.98 eV/atom (blue) to −3.9 eV/atom (red), in (a, c, e), and according to their local structural environment in (b, d, f). In the latter coloring scheme, the atoms with local fcc surroundings are colored green, atoms with local hexagonal close packed (hcp) surroundings are colored red, while all other atoms are colored gray. In water, the oxygen and hydrogen atoms are colored brown and light gray, respectively.

The evolution of the electron and lattice temperatures in the nanoparticles irradiated at a deposited energy density of ε = 0.5 eV/atom is shown in Figure 11a. The laser excitation of the conduction band electrons, described through the source term *S*_L_(*t*) in eq 3, leads to a sharp increase in *T*_e_ that reaches the maximum level of about 5500 K at 28 ps, shortly after the time the laser pulse reaches its peak intensity. The electron temperature then decreases due to electron–phonon equilibration, which is largely completed by about 55 ps. The energy transfer from the electrons leads to increase in *T*_l_, which reaches its maximum level of 1318 K at 38 ps, before the electron–phonon equilibration is completed. The lattice temperature then decreases due to the heat transfer to the surrounding water and reaches the level of 500 K by the time of 100 ps. We note that the energy density of 0.5 eV/atom is higher than the energy density required for heating of bulk Au from 300 K to *T*_m_^EAM^ and then completely meting it at *T*_m_^EAM^, 0.43 eV/atom.^20,112^ The reason the nanoparticle does not melt at ε = 0.5 eV/atom is the rapid energy transfer to the water environment that proceeds simultaneously with the energy transfer from the excited electrons. Indeed, a substantial heating of water by the time of 50 ps, i.e., close to the end of the electron–phonon equilibration (Figure 11a), is evidenced by the corresponding water temperature profile shown in Figure 11d.

It is notable that the solution of the continuum model, eqs 3–5, is in good quantitative agreement with the results of the TTM-MD modeling. The lattice temperature evolution predicted by the continuum model and shown in Figure 11a by the orange curve overlaps with the red curve, showing the result of the atomistic TTM-MD modeling. The water temperature profiles obtained with the continuum model and plotted in Figure 11d for 50 and 100 ps also describe reasonably well the data points from the atomistic modeling. This good agreement suggests that the parametrization of the model based on the steady state heat transfer simulations reported in Section 2 is applicable for the description of transient phenomena of laser heating and cooling of small Au nanoparticles.

At a higher deposited energy density of ε = 0.65 eV/atom, partial melting of the nanoparticle is predicted in both TTM-MD and continuum modeling. In the latter, partial melting is evidenced by the flat top of the lattice temperature profile in Figure 11b. According to the model assumption, as soon as the equilibrium melting temperature is reached, the additional energy supply from the hot electrons increases the molten fraction of the nanoparticle at a fixed temperature of *T*_l_ = *T*_m_^EAM^. In the atomistic modeling, the melting proceeds under nonequilibrium conditions of superheating above the equilibrium melting temperature, *T*_l_ > *T*_m_^EAM^, realized during a brief time interval from 30 to 48 ps. The maximum superheating in this simulation, *T*_l_ = 1475 K = 1.12*T*_m_^EAM^ is still below the level required for the onset of homogeneous melting, which is predicted to be about 1.25*T*_m_ for Au.^113,114^ Thus, the melting proceeds through the heterogeneous liquid phase nucleation at the surface of the nanoparticle and the propagation of the melting front toward the nanoparticle center. By the time when *T*_l_ gets below *T*_m_^EAM^, the melting is still incomplete, as can be seen from the snapshot shown for 48 ps in Figure 12c. In the snapshots colored by the potential energy of Au atoms, the color scale is chosen so that the solid and molten phases are visually distinct due to the contribution of the latent heat of melting to the latter. It is apparent that the central core of the nanoparticle remains crystalline at 48 ps. Since *T*_l_ < *T*_m_^EAM^ after 48 ps, melting turns into solidification that proceeds through the propagation of the liquid–crystal interface toward the surface of the nanoparticle. The velocity of the solidification front increases with increasing undercooling below *T*_m_^EAM^,^115–117^ and the solidification is completed by 110 ps, i.e., only 85 ps after the time when the laser pulse intensity reaches its maximum. The small shoulder on the lattice temperature curve predicted in the TTM-MD simulation and shown in Figure 11b is the result of the release of the latent heat of melting during the solidification.

The rapid solidification results in the generation of crystal defects, as can be seen from Figure 12d, where the atoms are colored by their local structural environment identified with the common neighbor analysis algorithm^118^ implemented in OVITO.^119^ The individual planes of atoms with local hcp surroundings (colored red) correspond to twin boundaries, while a stack of red planes corresponds to a small platelet of the metastable hcp structure.

Further increase in the deposited energy density to ε = 0.7 eV/atom results in the complete melting of the nanoparticle by 44 ps, as can be seen from the corresponding snapshot shown in Figure 12e. The complete melting is also predicted by the continuum model, where the little bump on the flat plateau of the temperature plot in Figure 11c corresponds to heating and cooling of the fully molten nanoparticle. While the temperature drops below *T*_m_^EAM^ at 48 ps in the TTM-MD simulation, the solidification is delayed by the absence of pre-existing crystalline nuclei. As a result, the nucleation and growth of the crystallites proceed under conditions of deep undercooling and yield highly distorted assembly of small nanocrystalline regions, Figure 12f. The energy stored by the defects in this structure corresponds to 0.050 eV/atom, which represents a substantial (37%) fraction of the latent heat of melting, Δ*H*_m_^EAM^ and can be converted to an equivalent temperature change of 189 K using the room temperature heat capacity of Au. The continuum model assumes that the heat of melting is fully released upon solidification, which explains the deviation of the temperature plots calculated with the continuum model from the data points predicted in the TTM-MD simulation, Figure 11c.

Overall, the results of the simulations of laser interactions with 7-nm Au nanoparticles confirm the ability of the continuum model to provide an adequate description of the evolution of temperature of the irradiated nanoparticles and to predict conditions for the onset of melting. Both atomistic and continuum simulations are performed for a finite size of the water shell surrounding the nanoparticle, although the effect of this finite size on the nanoparticle cooling is relatively small due to the large 72-fold difference in the total heat capacities of the water shell and the nanoparticle. This difference is reflected in a moderate heating of water observed by the end of the simulations in Figure 11d,e,f.

#### 3.2.2 Laser Interaction with 20-nm Au Nanoparticles

The increase in nanoparticle diameter from 7 to 20-nm gives rise to distinct features in the lattice temperature profiles, illustrated in Figure 13a for a simulation performed at a deposited laser energy of 0.3 eV/atom, below the melting threshold. The profiles for the central spherical core region and eight concentric layers within the nanoparticle begin to diverge during the laser energy deposition, and the temperature differences between layers diminish only gradually during cooling. The outer layer of the nanoparticle remains the coldest, while the central core attains and retains the highest temperature during the simulation. The establishment of the temperature gradient within the nanoparticle can be attributed to more efficient heat transfer from the outer layers of the nanoparticle directly adjacent to the cold water environment. A continuum-level description of the interfacial heat transfer, however, is far from being straightforward.

**Figure 13:**
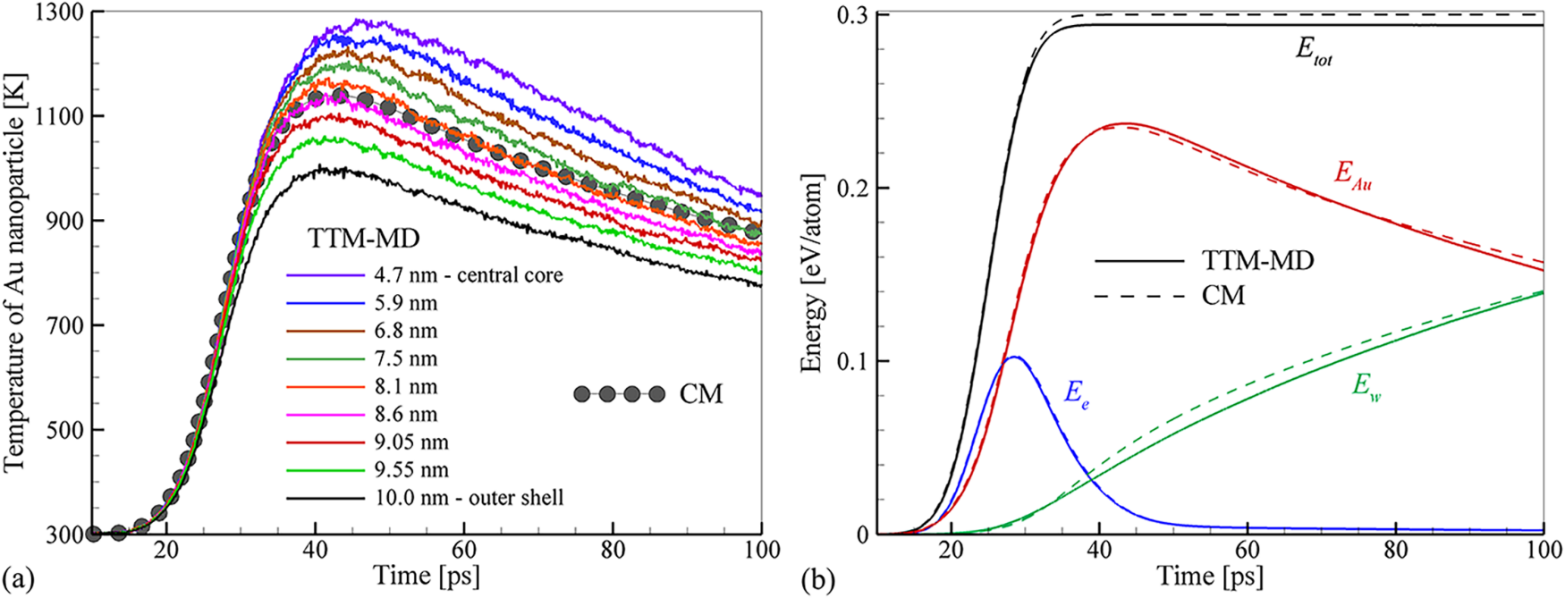
Evolution of lattice temperature (a) and different energy components (b) in atomistic and continuum simulations of a 20-nm Au nanoparticle irradiated in water by a 10 ps laser pulse at an absorbed energy density of ε = 0.3 eV/atom, below the threshold for the onset of melting. Results of TTM-MD simulations are compared with predictions of a continuum model (CM) based on numerical integration of eqs 3–5. In (a), TTM-MD temperature profiles are plotted for nine regions of the nanoparticle: a central core and eight concentric shells of approximately equal volume, labeled by their outer radius in the initial nanoparticle. Energy components in (b) are the total absorbed energy *E*_tot_, electron thermal energy *E*_e_, lattice thermal energy *E*_Au_, and energy transferred to water, *E*_w_. The laser pulse peak intensity occurs at 2.5τ_L_ = 25 ps.

Some of these challenges became evident in initial calculations with a model that defined the heat flux based on the temperature of the nanoparticle’s outer layer, i.e., *Q*_r_^int^ = (*T*_Au_(*R*) – *T*_w_(*R*))*h*_*K*_(*T*_Au_,*Q*_r_^int^,*R*), and removed all energy transferred by the interfacial heat flux across the gold/water interface from this layer. Because of the large flux at the early stage of cooling, the outer layer temperature dropped to an unrealistically low level, which reduced the heat flux and led to a substantial underestimation of the nanoparticle cooling rate with respect to the prediction of the atomistic model. The resupply of energy to the outer layer by electron heat transfer was constrained by the finite electron–phonon equilibration time and could not compensate for the energy loss to the surrounding water. Moreover, the predicted cooling rate in this approach was sensitive to the spatial discretization chosen for the nanoparticle. These issues are related to the faulty assumption that only the surface layer of the nanoparticle participates in the heat exchange with water. Indeed, this assumption does not capture the actual mechanism of the interfacial heat transfer, as the heat carriers (phonons) are not localized down to the atomic level.

The results of the atomistic modeling suggest that the interfacial heat transfer removes thermal energy from different parts of the nanoparticle, albeit to different extent. In the absence of a reliable theoretical model for the nanoscale interfacial heat transfer, the present study adopts a simplified approach that neglects the temperature gradient within the nanoparticle, representing its thermal state by a single lattice temperature, *T*_Au_. This average temperature is higher than the actual temperature of the Au–water interface in the atomistic model, leading to an overestimation of *h*_*K*_ taken from steady-state NEMD simulations, where a uniform temperature is enforced throughout the nanoparticle thickness (e.g., Figure 4). To account for the effect of the temperature gradient, the value of *h*_*K*_ is scaled by a correction factor ω, i.e., the interfacial thermal conductance of *h*_*K*_^′^ = ω*h*_*K*_(*T*_Au_,*Q*_r_^int^,*R*) is used for the 20-nm nanoparticle. A value of ω = 0.75, chosen to match the total energy transferred to water by 100 ps, provides a good overall description of the evolution of different energy components in the nanoparticle–water system (Figure 13b) as well as the average nanoparticle temperature (Figure 13a).

A small discrepancy in the total energies of the system in the atomistic and continuum models, shown by the black solid and dashed curves in Figure 13b, respectively, is related to the energy carried away by a spherical pressure wave generated during nanoparticle expansion in response to laser heating in the atomistic model. The pressure wave propagates through the water layer and exits the computational domain via a spherical pressure-transmitting boundary. The energy removed by the wave amounts to about 2% of the energy deposited by the laser pulse, which is significantly lower than the 12% predicted in simulations of laser fragmentation of a 20-nm Au nanoparticle at a 12-fold higher deposited energy of 3.6 eV/atom,^21^ but is comparable to ∼2% of the deposited energy predicted for a planar pressure wave generated in a bulk Ni target irradiated by a 1 ps laser pulse below the melting threshold.^120^

As the deposited laser energy increases, the irradiated nanoparticle undergoes partial or complete melting, followed by solidification. These processes are illustrated in Figure 14a,c,e by cross-sectional snapshots of atomic configurations colored by atomic potential energy. At 0.45 eV/atom, multiple small liquid nuclei transiently form in the central part of the nanoparticle (Figure 14a), where the temperature is highest and pressure oscillations further reduce lattice stability against melting.^121^ As the nanoparticle cools below the melting point, the liquid regions disappear by 100 ps, leaving behind a high density of point defects (Figure 14b). In particular, at 100 ps, i.e., immediately after complete resolidification, the nanoparticle contains 12 vacancies and 9 interstitials in ⟨100⟩ dumbbell configurations. Most of these point defects (86%) are located within a 7-nm distance from the center of the nanoparticle.

**Figure 14:**
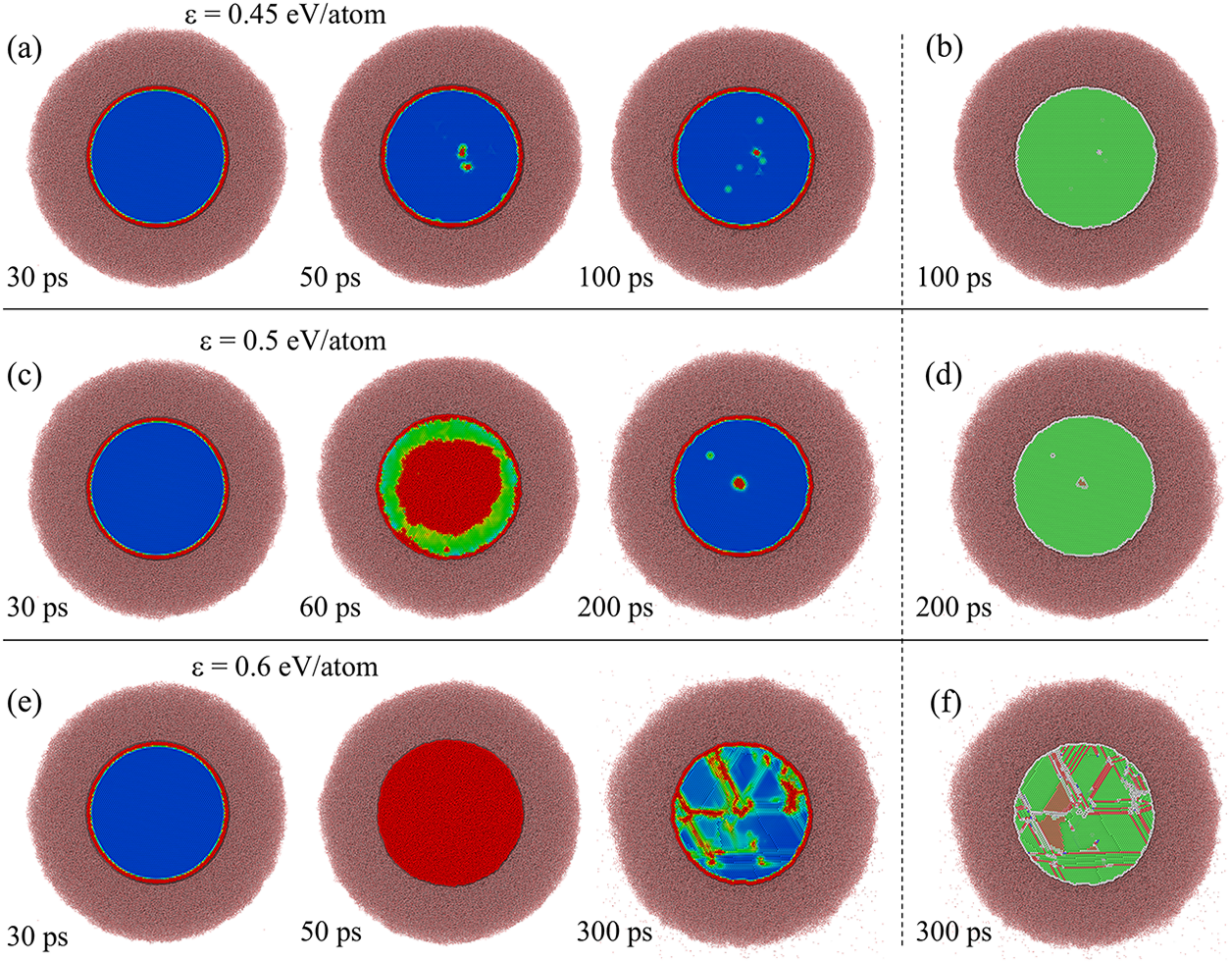
Cross sections of atomic configurations predicted in TTM-MD simulations of 20-nm Au nanoparticles irradiated in water by 10 ps laser pulses at absorbed energy densities of 0.45 (a, b), 0.5 (c, d), and 0.6 eV/atom (e, f). The Au nanoparticles are quenched to 0 K prior to making the plots and are colored by their potential energy averaged over neighbors within a 3.5 Å radius, ranging from −3.98 eV/atom (blue) to −3.9 eV/atom (red), in (a, c, e), and according to their local structural environment in (b, d, f). In the latter coloring scheme, the atoms with local fcc surroundings are colored green, atoms with local hcp surroundings are colored red, while all other atoms are colored gray. In water, the oxygen and hydrogen atoms are colored brown and light gray, respectively.

At a higher energy of 0.5 eV/atom, the liquid nuclei coalesce into a central molten region surrounded by a solid shell (Figure 14c). The fraction of the molten phase reaches its maximum by 60 ps and gradually decreases as heat is transferred to the water. Solidification completes by 192 ps, when the quasi-spherical solidification front collapses and produces a cluster of point defects arranged into a prismatic dislocation loop in the nanoparticle center (Figure 14d).

Further increase in the deposited energy density to 0.6 eV/atom leads to complete melting, followed by resolidification that proceeds under conditions of deep undercooling and is complete by about 300 ps (Figure 14e). Similar to the smaller 7-nm nanoparticle (Figure 12f), the complete melting and resolidification produces a structure with a high density of planar crystal defects (Figure 14f), although the defect density is smaller for the larger 20-nm nanoparticle due to the lower cooling rate leading to the solidification (*cf*. Figures 11c and 15a).

**Figure 15:**
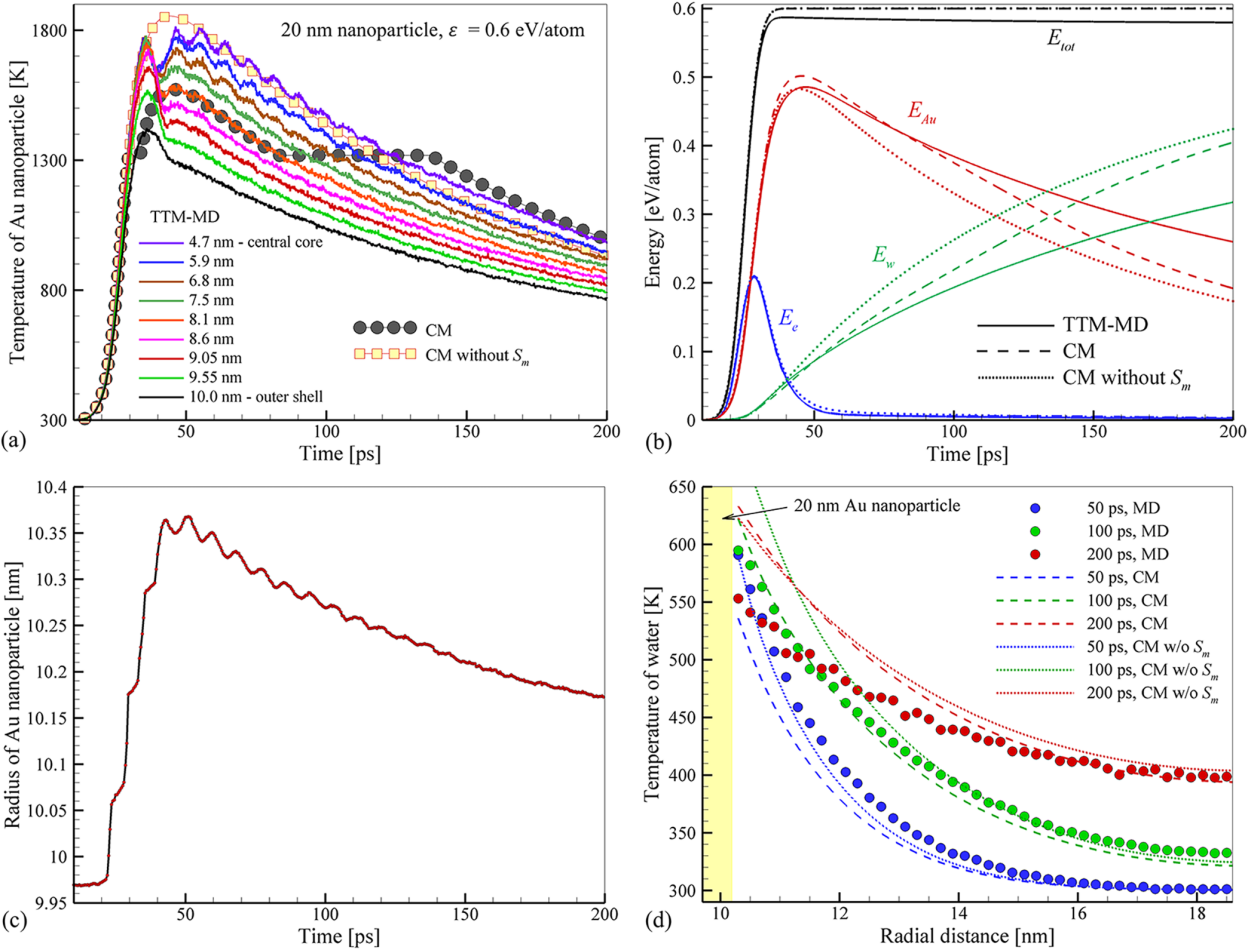
Evolution of lattice temperature (a), energy components (b), nanoparticle radius (c), and water temperature (d) in atomistic and continuum simulations of a 20-nm Au nanoparticle irradiated in water by a 10 ps laser pulse at an absorbed energy density of ε = 0.6 eV/atom, above the threshold for complete melting of the nanoparticle. Results of TTM-MD simulations are compared with predictions of a continuum model (CM) based on numerical integration of eqs 3–5. The CM solution neglecting the energy transformations due to melting and solidification, i.e., with the term *S*_m_(*T*_l_, *f*_l_) omitted in eq 4, is shown in (a, b, d) alongside the predictions of the complete CM. In (a), TTM-MD temperature profiles are plotted for nine regions of the nanoparticle: a central core and eight concentric shells of approximately equal volume, labeled by their outer radius in the initial nanoparticle. Energy components in (b) are the total absorbed energy *E*_tot_, electron thermal energy *E*_e_, lattice thermal energy *E*_Au_, and energy transferred to water, *E*_w_. The laser pulse peak intensity occurs at 2.5τ_L_ = 25 ps.

The generation of crystal defects can be related to earlier computational^18–23^ and experimental^11,12,17,23^ reports on the formation of defect-rich nanoparticles in laser-assisted nanoparticle synthesis and processing, where nanodroplets produced by laser ablation or fragmentation in liquids undergo rapid cooling and solidification. The ability of laser processing to produce defect-rich nanoparticles is of high practical importance, as the presence of crystal defects has been demonstrated to result in a strong enhancement of nanoparticle catalytic activity.^10–17^

At energy densities that induce melting of 20-nm nanoparticles, the interfacial heat transfer conditions lie well outside the range explored in the NEMD simulations of Section 2.3, making the applicability of eq 2 questionable. Moreover, the stability condition for the solution with *h*_*K*_^′^ = ω*h*_*K*_(*T*_Au_,*Q*_r_^int^ *,R*) described by eq 2, Δ*T* < (ω*b*)^−1^ = 738 K, is violated in the simulation performed at ε = 0.6 eV/atom. Therefore, the continuum-level calculations for this energy density are performed with a constant interfacial conductance, *h*_*K*_^′^ = 111 MWm^–2^ K^–1^, chosen to reproduce the total energy transferred to water by 50 ps in the corresponding atomistic simulation. The results show reasonable overall semiquantitative agreement with atomistic modeling (Figure 15). In particular, the continuum model predicts complete melting of the nanoparticle by 33 ps, onset of resolidification at 82 ps, and completion of resolidification by 135 ps (Figure 15a). The evolution of the energy components (Figure 15b) and the water temperature distributions (Figure 15d) are close to the atomistic results at early times but diverge increasingly as the simulation progresses. This divergence reflects an overestimation of the interfacial conductance at later stages of nanoparticle cooling, when a constant *h*_*K*_^′^ is assumed.

One notable prediction of the continuum simulation is that the nanoparticle temperature after complete solidification lies above the upper bound of the temperature profiles from the atomistic simulation (Figure 15a). This result may seem counterintuitive since the nanoparticle energy at the same time is underestimated by the continuum model (Figure 15d). In the atomistic simulation, the lower temperature yet higher energy of the nanoparticle arises from the incomplete release of latent heat during resolidification. A fraction of this energy is stored in the defects formed during rapid solidification, as illustrated in the cross-sectional views of the nanoparticles shown in Figure 14e,f for 300 ps. The energy associated with these defect structures is 0.0147 eV/atom (11% of Δ*H*_m_^EAM^), which corresponds to an equivalent temperature change of 56 K when converted by using the room temperature heat capacity of Au. This contribution is absent in the continuum model, where the latent heat of melting is assumed to be fully released as thermal energy upon solidification.

Another important observation is the substantial increase in the water temperature at the outer boundary of the computational domain (e.g., see profile shown for 200 ps in Figure 15d). The trend toward the equalization of temperature throughout the water shell indicates that the finite size of the domain significantly affects the evolution of the temperature gradient in water and, consequently, the nanoparticle cooling. Unlike the 7-nm nanoparticle discussed in Section 3.2.1, where the water shell has a total heat capacity 72 times greater than that of the Au nanoparticle, this ratio decreases to 9.3 for the 20-nm nanoparticle. The smaller ratio leads to a more pronounced heat accumulation in the water shell. Since increasing the water shell thickness is computationally expensive, the next section introduces an alternative, computationally efficient approach to representing heat transfer from the nanoparticle to the surrounding water.

### 3.3 Atomistic-Continuum Model of Nanoparticles in “Implicit” Water Environment

The analysis of the results of the atomistic and continuum-level simulations of laser-induced heating and cooling of Au nanoparticles in water reveals both the advantages and the limitations of the two approaches. While the atomistic model provides a realistic description of the interfacial heat transfer and thermal energy dissipation into the water environment, the high computational cost of the simulations makes it difficult to make the size of the water shell sufficiently large for a realistic representation of the natural evolution of the temperature field, particularly around large nanoparticles. The continuum model is computationally efficient and suitable for the exploration of the space of irradiation parameters but suffers from several assumptions that limit the predictive power of the simulations. In this section, a hybrid atomistic-continuum model that combines the strengths of the two models is suggested.

The hybrid model combines a continuum representation of the heat transfer in the water environment with atomistic modeling of the nanoparticle response to laser excitation. The heat diffusion in the water environment is still described using eq 5 coupled to the nanoparticle through the boundary condition *T*_w_(*R*) = *T*_Au_ – *Q*_r_^int^/*h*_*K*_, defined at the nanoparticle–water interface, i.e., at *r = R*. The nanoparticle temperature *T*_Au_ is taken here as the average temperature of the nanoparticle. At the outer boundary of the computational domain, adiabatic boundary condition is applied. Since the integration of eq 5 has a negligible computational cost, the boundary is placed at 100 nm, which is sufficiently far for eliminating any effect of the boundary condition on the temperature evolution in the vicinity of the nanoparticle.

The nanoparticle is represented with the TTM-MD model, similar to the atomistic simulations described in Section 3.2. One essential difference is that the hybrid model is implemented in an in-house code,^21,105^ allowing for a refined description of the energy redistribution within the nanoparticle. Instead of defining a single (average) lattice temperature for the whole nanoparticle, as in Section 3.2.1 for the 7-nm nanoparticle, or defining separate lattice temperatures for a central spherical core region and eight concentric layers, as in Section 3.2.2 for the 20-nm nanoparticle, the local lattice temperature is now defined at the position of each atom. The temperature at the position of atom *i* is calculated by averaging the kinetic energies of all *N*_*i*_^c^ atoms located within a distance *R*_c_ from atom *i, T̅*_*l*_^*i*^ = 2*K̅*_*i*_^th^/3*k*_B_, where *k*_B_ is the Boltzmann constant and the average kinetic energy is defined as

6 $${\mathrm{K̅}}_{i}^{\mathrm{th}}=\frac{1}{2\left(N_{i}^{c}+1\right)}\sum_{j}^{N_{i}^{c}+1}m_{j}{\left({\mathrm{v⃗}}_{j}-{\mathrm{v⃗}}_{i}^{c}\right)}^{2}$$

where *m*_*j*_ and *v⃗*_*j*_ are the mass and velocity of an atom *j*, the summation is over all atoms located within a distance *R*_c_ from atom *i* as well as the atom *i* itself, and *v⃗*_*i*_^c^ is the center-of-mass velocity of these *N*_*i*_^c^ + 1 atoms. The subtraction of the center-of-mass velocity ensures that the temperature is defined based on thermal velocities of atoms and does not include contributions from collective atomic motion, e.g., due to the laser-induced elastic oscillations of a nanoparticle.

The thermal motion of atoms is coupled to a single pool of electron energy described by eq 3. The energy exchange between the electron and lattice subsystems is realized by adding coupling terms to the MD equations of motion and eq 3 for the electrons. On the MD side, the coupling term also accounts for the energy transfer by the heat flux *Q*_r_^int^ through the nanoparticle–water interface, so that the modified equations of motion take the following form

7 $$m_{i}\left(d^{2}{\mathrm{r⃗}}_{i}/dt^{2}\right)={\mathrm{F⃗}}_{i}+\xi m_{i}{\mathrm{v⃗}}_{i}^{th},,\mathrm{where}\;\xi =\left[GV_{a}\left(T_{e}-{\mathrm{T̅}}_{l}^{i}\right)-4\pi R^{2}Q_{r}^{int}/N_{np}\right]/2{\mathrm{K̅}}_{i}^{th}$$

where *r⃗*_*i*_ are the position of an atom *i*, *F⃗*_*i*_ is the force acting on atom *i* due to the interatomic interactions, *v⃗*_i_^th^ = *v⃗*_*i*_ – *v⃗*_*i*_^c^ is the velocity of thermal motion of atom *i*, *N*_np_ is the total number of atoms in the nanoparticle, and *V*_a_ is the average volume per atom. In the expression for the coefficient ξ, the first term accounts for the energy exchange with the electron subsystem,^105^ while the second term corresponds to the heat removed from the nanoparticle through the interface with water environment.

The definition of local temperature adopted in the present model relies on averaging over a sufficiently large number of atoms in eq 6, so that the values of *K̅*_*i*_^th^ and *T̅*_*l*_^*i*^ are not affected by statistical noise. The choice of *R*_c_ = 9.875 Å (interatomic potential cutoff distance plus 3 Å) is found to yield an average value of *N*_*i*_^c^ + 1 equal to about 218 atoms at room temperature and about 208 atoms at *T*_Au_ = 1.2*T*_m_^EAM^ for a 20-nm Au nanoparticle. The averaging over these numbers of atoms provides reliable estimates of the local temperature values.

The advantage of resolving the local lattice temperature distribution within the nanoparticle is that any temperature heterogeneities, e.g., those arising from heat release or absorption during local melting or solidification, trigger energy redistribution controlled by the strength of electron–phonon coupling. This redistribution occurs on a time scale substantially shorter than that provided by phononic (vibrational) thermal conductivity, which is naturally captured in MD simulations. As a result, electron-assisted temperature equilibration is adequately represented in the hybrid atomistic-continuum model.

The performance of the hybrid model with “implicit” water representation is tested in three simulations of laser-induced partial or complete melting of 7 and 20-nm nanoparticles. Similar to simulations discussed in Section 3.2, the nanoparticles are irradiated by 10 ps laser pulses, and the laser energy deposition is implemented through the source term in eq 3. The nanoparticles are equilibrated at 300 K for 300 ps prior to the simulation of laser excitation. To enable direct comparison with continuum simulations discussed above, the interfacial conductance is described by eq 1 for the 7-nm nanoparticle, while a constant value of 111 MW m^–2^ K^–1^ is used for the 20-nm nanoparticle.

The results of two simulations performed for the 7-nm nanoparticle are illustrated by cross sections of atomic configurations and temperature fields in the surrounding water in Figure 16. The processes of partial (Figure 16a) or complete (Figure 16c) melting and resolidification are consistent with those predicted in the fully atomistic simulations and illustrated in Figure 12c,e, respectively. The final configuration produced by the partial melting and resolidification in Figure 16b features several planar defects (twins and stacking faults) and is similar to that generated in the corresponding fully atomistic simulation and shown in Figure 12d. In the case of complete melting, Figure 16c, the solidification produces a much higher density of crystal defects (Figure 16d), which again matches the observations from the fully atomistic simulation (Figure 12f). The temperature of water adjacent to the nanoparticle irradiated at ε = 0.7 eV/atom reaches the maximum level of 539 K at 48 ps, but rapidly drops as the heat transferred from the nanoparticle dissipates through the radial heat conduction. The maximum temperature and the subsequent rapid cooling of water are similar to those predicted in the fully atomistic simulation performed at ε = 0.7 eV/atom and shown in Figure 11f.

**Figure 16:**
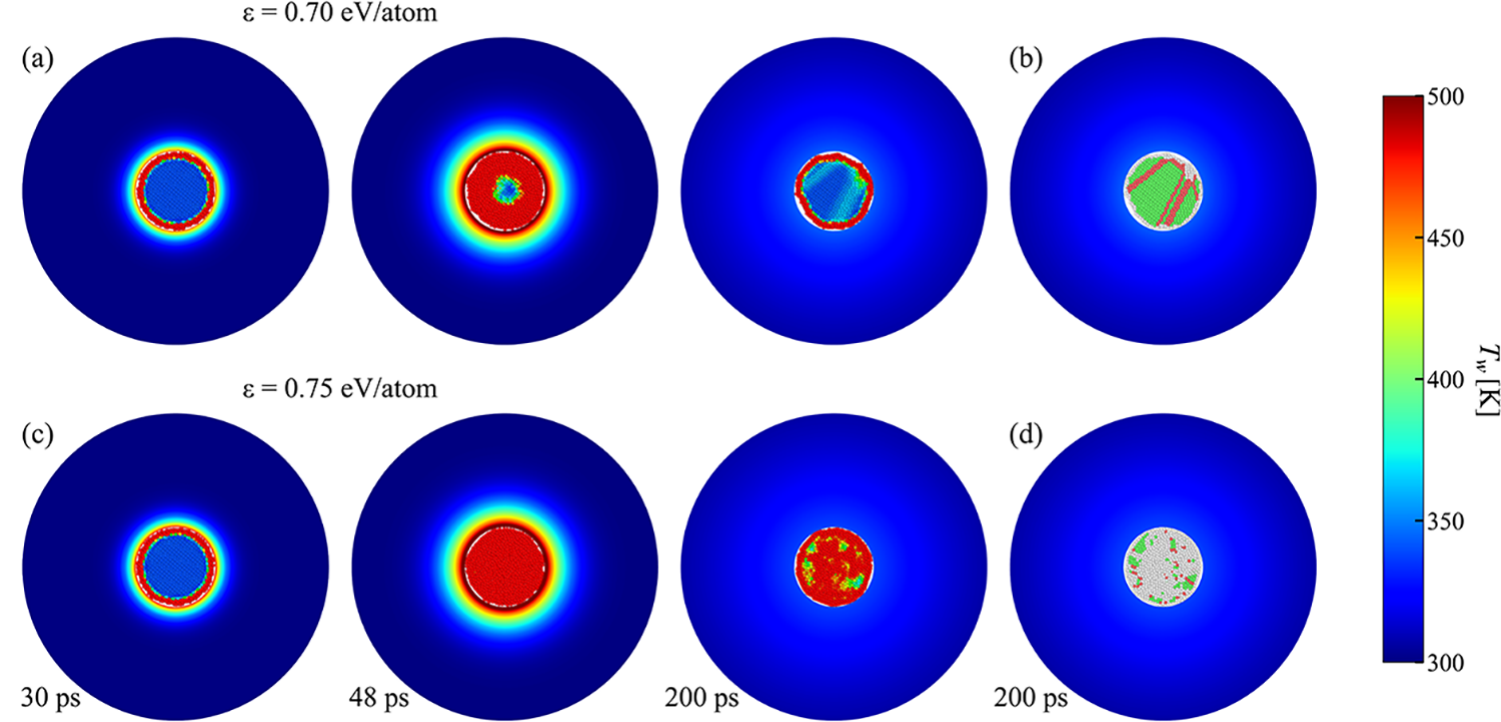
Cross sections of atomic configurations predicted in TTM-MD simulations of 7-nm Au nanoparticles coupled to an “implicit” water domain. The nanoparticles are irradiated by 10 ps laser pulses at absorbed energy densities of 0.7 (a, b) and 0.75 eV/atom (c, d). The atomic configurations are quenched to 0 K prior to making the plots. In (a, c), Au atoms are colored by their potential energy averaged over neighbors within a 3.5 Å radius, ranging from −3.98 eV/atom (blue) to −3.9 eV/atom (red). In (b, d), Au atoms are colored according to their local structural environment, so that atoms with local fcc surroundings are green, and atoms with local hcp surroundings are red, while all other atoms are gray. Only a 10-nm-thick part of the “implicit” water domain is shown and is colored by the temperature.

One quantitative difference between the predictions of the two models is a moderate (by less than 0.05 eV/atom) shift of the threshold energy for the transition from partial to complete melting to higher energy density in the model adopting the “implicit” water description. This shift can be attributed to the uniform extraction of energy transferred to water from the nanoparticle assumed in eq 7. The fully atomistic model predicts the generation of a temperature gradient within the nanoparticle, which is particularly apparent for the 20-nm nanoparticle (Figures 13a and 15a) but is also present in the smaller 7-nm nanoparticle. The gradient leads to a higher temperature of the core of the nanoparticle and results in the complete melting in the fully atomistic simulation at an energy density of 0.7 eV/atom, while the nanoparticle is only partially melted in the simulation with implicit water (cf. Figures 12e,f and 16a,b).

For the 20-nm Au nanoparticle, the simulation illustrated in Figure 17 is performed for the deposited energy density of 0.6 eV/atom, which corresponds to the complete melting of the nanoparticle followed by resolidification. The results are similar to those obtained with the fully atomistic model at the same energy density and illustrated in Figures 14e,f and 15. The average nanoparticle temperature profiles predicted by the two models and shown by the solid red and dashed green curves in Figure 17a are almost identical up to a time of about 100 ps. By this time, heat transfer extends to the outer boundary of the 9 nm water shell used in the atomistic simulation (Figure 15d), elevating the temperature at the boundary to about 335 K. Further heat transfer results in artificial flattening of the radial temperature profile due to the finite-size effect, leading to the divergence of the temperature profiles in Figure 17a. The reduced temperature gradient in the fully atomistic model (cf. profiles for 200 ps in Figures 15d and 17b) lowers the conductive heat flux away from the nanoparticle and reduces the nanoparticle cooling rate. By the time of solidification that proceeds at around 300 ps (as evidenced by the slight shoulder of the temperature profile shown by the red line in Figure 17a), the two curves diverge by 74 K. The “implicit” water model extends up to 100 nm from the surface of the nanoparticle and is free of finite-size artifacts, leading to the faster cooling and stronger undercooling below the equilibrium melting temperature at the time of resolidification. As a result, the structure of the solidified nanoparticle features a higher level of structural disorder, with smaller and more numerous grains and twinned domains (cf. Figures 14f and 17d). Note that the large number of gray atoms in Figure 17d does not imply the presence of an amorphous structure in the nanoparticle. The gray atoms correspond to the grain boundary regions and highly distorted crystal structure. The latter can also be identified as crystalline (green or red) atoms, depending on the tolerance to unit cell distortion in the crystal structure analysis algorithm.

**Figure 17:**
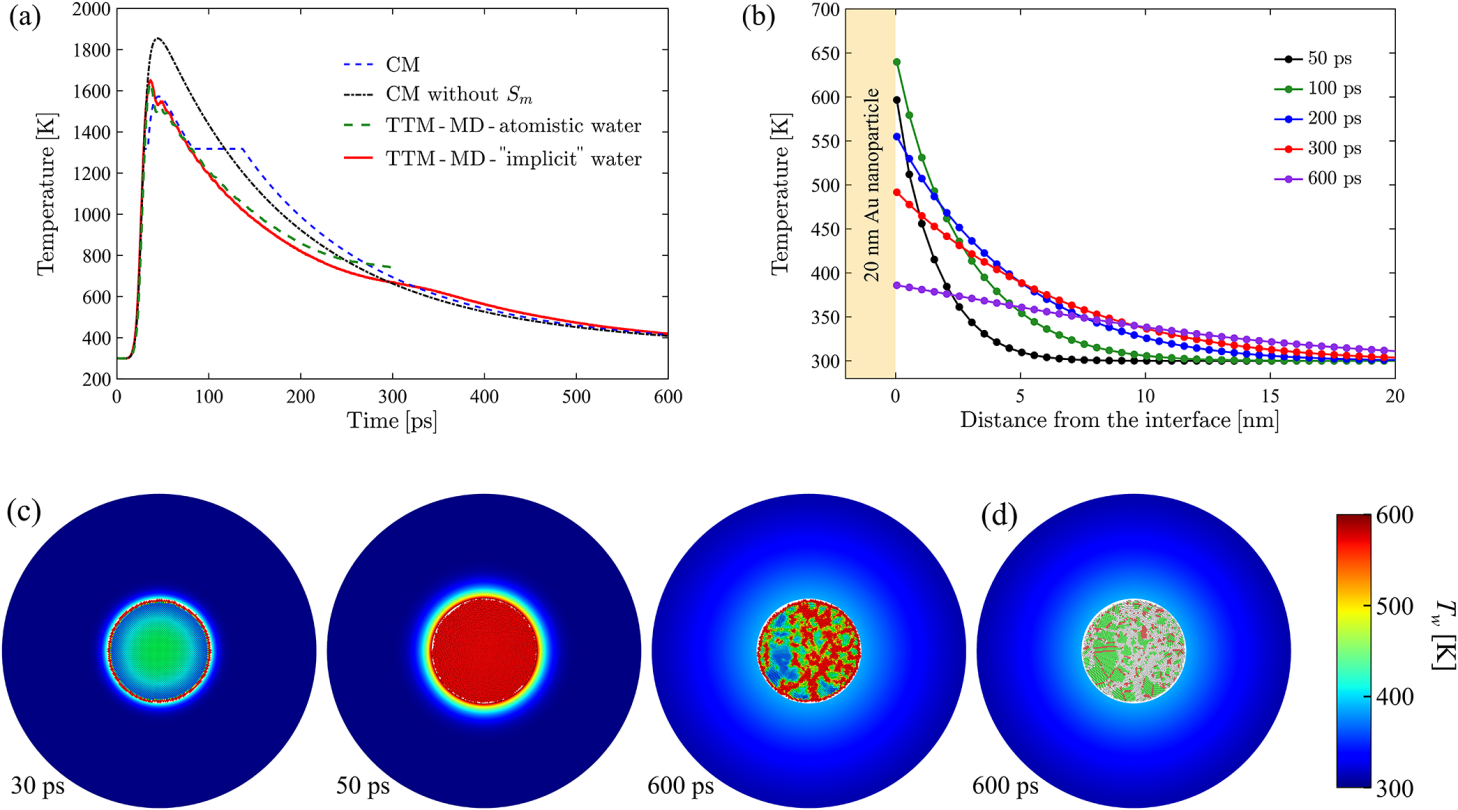
Evolution of temperature and atomic configurations predicted in a TTM-MD simulation of a 20-nm Au nanoparticle coupled to an “implicit” water domain and irradiated by a 10 ps laser pulse at an absorbed energy density of 0.6 eV/atom. In (a), the temperature of the Au nanoparticle is shown by the red curve and is compared with predictions of continuum and fully atomistic models discussed in previous sections. In (b), the temperature profiles in the water environment are shown for five moments of time after the start of the simulation (the laser pulse peak intensity occurs at 25 ps). In (c, d) cross sections of atomic configurations predicted in the simulation are shown along with a 20-nm-thick part of the surrounding water environment colored by temperature. The atomic configurations are quenched to 0 K prior to making the plots. In (c), Au atoms are colored by their potential energy averaged over neighbors within a 3.5 Å radius, ranging from −3.98 eV/atom (blue) to −3.9 eV/atom (red). In (d), Au atoms are colored according to their local structural environment, so that atoms with local fcc surroundings are green, atoms with local hcp surroundings are red, while all other atoms are gray.

Overall, the comparison of the computational models suggests that the hybrid atomistic-continuum model with “implicit” water representation can provide a realistic description of the laser-induced structural modification of colloidal nanoparticles at a small fraction of the computational cost of the fully atomistic model. For example, a simulation of laser melting and resolidification of a 20-nm nanoparticle for a time of 600 ps takes about 7 wall-clock hours (about 450 core-hours) on half a compute node of the Anvil supercomputer (AMD EPYC 7763 64-core processor) with the hybrid model, while an equivalent fully atomistic simulation has about 143 times higher computational cost. Moreover, the small thickness of the water shell (9 nm) included in the fully atomistic simulation is inadequate for modeling the heat dissipation from 20-nm and larger nanoparticles. Indeed, the continuum and hybrid simulations show that the “heat affected zone,” defined as the thickness of a water shell where the temperature exceeds 310 K, extends to about 20 nm away from the nanoparticle–water interface by 600 ps in the simulation illustrated by Figure 17. The explicit representation of a 20-nm water shell around a 20-nm nanoparticle would include more than 3.6 million water molecules, making the simulation rather expensive. We also note that while the continuum-level models cannot be used for analysis of the melting/solidification kinetics and structural modification of nanoparticles, they are still suitable for a quick evaluation of the overall cooling time of the nanoparticles, as can be seen from predictions of the continuum models shown by blue dashed and black dashed-dotted lines in Figure 17a.

Finally, we note that the applicability of the models discussed in this paper is limited to conditions that do not result in the formation of transient nanobubbles around the laser-heated nanoparticles.^18,20,33,35–40,59^ The results of our fully atomistic simulations suggest that the robust heat transfer in the course of nanoparticle cooling and solidification is sustained by the suppression of nanobubble formation by the high curvature of the interface, even under conditions when the gold temperature exceeds the critical temperature of water by more than a factor of 2. Thus, no nanobubble formation is observed for 20 nm or smaller nanoparticles at energy densities extending up to the threshold for complete melting of the nanoparticles. Further increase in the energy deposited by the laser pulse or increase in the radius of the nanoparticle, however, may lead to the transient formation of a nanobubble, with important implications on the interfacial heat transfer and cooling of the nanoparticle.^18,20,122^ Earlier simulations performed for 20-nm Au nanoparticles with a coarse-grained representation of water environment suggest that the lowest energy density leading to the formation of a nanobubble is 1.2 eV/atom, which corresponds to the onset of evaporation of Au atoms from the surface of the nanoparticle.^20^ The maximum temperature reached by the Au nanoparticle by the end of the laser pulse in this case is 3300 K, and the nanobubble only expands to about 4 nm by 100 ps after the laser pulse, collapses by about 300 ps, and then exhibits several weak rebounds until about 700 ps. An adequate representation of the generation of nanobubbles in a fully atomistic model is computationally expensive, calling for coarse graining of the MD representation of the liquid environment^21^ or coupling of the MD to a hydrodynamic model.

## 4 Summary

The processes controlling the heat transfer from colloidal gold nanoparticles rapidly heated by a short pulse laser irradiation to the surrounding water environment are investigated in a multiscale computational study that combines nonequilibrium molecular dynamics with continuum-level modeling. First, the thermal conductivity of water is examined in NEMD simulations, confirming the ability of the SPC/E potential to provide a reliable quantitative description of thermal conductivity over a broad range of temperatures, including the sharp conductivity drop near the critical temperature.

Next, the temperature dependence of the interfacial thermal conductance of planar Au–water interfaces is analyzed, revealing the existence of two regimes: a gradual decrease of the conductance from 110 to ∼80 MWm^–2^ K^–1^ with increasing temperature at low Au temperatures (400 to 700 K) and a sharp decline down to less than 20 MWm^–2^ K^–1^ as *T*_Au_ exceeds 1200 K. The transition from the first to the second regime corresponds to the formation of a low-density layer of supercritical water, where *T*_w_ > *T*_c_^SPC/E^. The combined/effective conductance of the Au–water interface and the supercritical layer is found to exhibit a discontinuous drop by about a factor of 2 upon the formation of the supercritical layer, suggesting a threshold-like dependence of the efficiency of the heat transfer through a planar Au–water interface on the thermodynamic state of the interfacial water.

Extending the NEMD simulations of steady-state heat transfer to 7- and 20-nm Au nanoparticles reveals that the interfacial curvature significantly enhances the interfacial thermal conductance and suppresses the formation of the supercritical layer. For 7-nm Au nanoparticles, the interfacial conductance is 20–30% higher compared to the case of a planar interface at low Au temperatures (*T*_Au_ < 700 K), and the supercritical layer does not form up to the maximum *T*_Au_ = 1400 K studied in the NEMD simulations with a water heat bath temperature of 300 K. When the supercritical layer does form (at *T*_Au_ ≥ 1000 K and *T*_w_^HB^ = 500 K), its thickness remains small (below 1 nm) up to *T*_Au_ = 1400 K, and the interfacial conductance stays substantially higher as compared to that of the planar interface. The suppression of the formation of low-density regions (or nanobubbles) around the nanoparticles plays a key role in sustaining the high interfacial thermal conductance at high temperatures. It is attributed to the compression produced by the Laplace pressure generated by a nanoscale diffuse interface between the supercritical and normal water surrounding the nanoparticle.

The atomistic insights into the dependence of thermal boundary conductance on Au temperature, interfacial heat flux, and curvature of the interface are incorporated into a continuum model that couples a two-temperature description of electron–phonon equilibration in laser-irradiated nanoparticles with the solution of the heat diffusion equation for the surrounding water. Validation of the continuum model against fully atomistic TTM-MD simulations of heating, melting, and resolidification of colloidal Au nanoparticles irradiated by 10 ps laser pulses demonstrates the ability of the model to provide an adequate description of the evolution of temperature of the irradiated nanoparticles and predict conditions for the onset of melting. In the regime of melting and resolidification, some deviations between the predictions of the two models are observed and related to the following deficiencies of the continuum model: (1) a simplistic representation of the melting and solidification processes that does not allow for superheating/undercooling of Au above/below the equilibrium melting temperature and (2) the assumption that the heat of melting is fully recovered upon the solidification, thus neglecting the energy stored by crystal defects generated in rapid nonequilibrium solidification.

To address these limitations, a hybrid atomistic-continuum model that combines a continuum treatment of the heat transfer in the water environment with atomistic modeling of the structural and phase transformations in the nanoparticle is designed and applied for simulation of laser-induced melting and resolidification of Au nanoparticles. The elimination of the atomistic water, which accounts for a major fraction of the computational cost in the fully atomistic model, makes the hybrid model more efficient and suitable for exploration of the irradiation parameter space. Moreover, the hybrid model is free of artifacts related to the finite thickness of the water shell in the fully atomistic model, as the solution of the heat diffusion equation can be extended to large distances at a negligible computational cost.

Simulations performed with the hybrid and fully atomistic models predict that Au nanoparticles irradiated by 10 ps laser pulses in water experience rapid cooling, on a time scale of ∼100 ps for 7 nm and several hundred ps for 20-nm nanoparticles. At sufficiently high laser energies, nanoparticles undergo complete melting, followed by resolidification under conditions of deep undercooling, producing nanoparticles with high densities of crystal defects. These findings provide a mechanistic basis for explaining the enhanced catalytic activity often reported for defect-rich nanoparticles synthesized or processed by laser irradiation in liquids.

The multiscale computational framework developed in this work, which combines atomistic and continuum heat-transfer modeling in both sequential and parallel modes, provides a robust foundation for simulating laser interactions with colloidal nanoparticles. Owing to its generality, the approach can be readily extended to nanoparticles of different types, sizes, and compositions, enabling broad applicability in future studies.

Overall, this study elucidates the interplay between interfacial conductance, nanoparticle curvature, and phase transformations during laser–nanoparticle interactions in liquids, advancing both fundamental understanding and modeling capabilities for laser synthesis and processing of colloidal nanoparticles.

## References

[1] Link S., Burda C., Nikoobakht B., El-Sayed M. A. (2000). Laser-induced shape
changes of colloidal gold nanorods using femtosecond and nanosecond
laser pulses. J. Phys. Chem. B.

[2] Wang H., Pyatenko A., Kawaguchi K., Li X., Swiatkowska-Warkocka Z., Koshizaki N. (2010). Selective
pulsed heating for the synthesis of semiconductor
and metal submicrometer spheres. Angew. Chem.,
Int. Ed..

[3] Hashimoto S., Werner D., Uwada T. (2012). Studies on the interaction of pulsed
lasers with plasmonic gold nanoparticles toward light manipulation,
heat management, and nanofabrication. J. Photochem.
Photobiol. C.

[4] Swiatkowska-Warkocka Z., Pyatenko A., Krok F., Jany B. R., Marszalek M. (2015). Synthesis
of new metastable nanoalloys of immiscible metals with a pulse laser
technique. Sci. Rep..

[5] Zhang D., Gökce B., Barcikowski S. (2017). Laser synthesis and processing of
colloids: Fundamentals and applications. Chem.
Rev..

[6] Ishikawa Y., Tsuji T., Sakaki S., Koshizaki N. (2023). Pulsed laser
melting in liquid for crystalline spherical submicrometer particle
fabrication – Mechanism, process control, and applications. Prog. Mater. Sci..

[7] Kim M., Lee J.-H., Nam J.-M. (2019). Plasmonic photothermal nanoparticles
for biomedical applications. Adv. Sci..

[8] Cui X., Ruan Q., Zhuo X., Xia X., Hu J., Fu R., Li Y., Wang J., Xu H. (2023). Photothermal nanomaterials:
A powerful light-to-heat converter. Chem. Rev..

[9] Sztandera K., Gorzkiewicz M., Klajnert-Maculewicz B. (2019). Gold nanoparticles in cancer treatment. Mol. Pharmaceutics.

[10] Zhang D., Liu J., Li P., Tian Z., Liang C. (2017). Recent advances in
surfactant-free, surface-charged, and defect-rich catalysts developed
by laser ablation and processing in liquids. ChemNanoMat.

[11] Li Z., Fu J.-Y., Feng Y., Dong C.-K., Liu H., Du X.-W. (2019). A silver
catalyst activated by stacking faults for the hydrogen evolution
reaction. Nat. Catal..

[12] Lin J.-Y., Xi C., Li Z., Feng Y., Wu D.-Y., Dong C.-K., Yao P., Liu H., Du X.-W. (2019). Lattice-strained palladium nanoparticles
as active catalysts for the oxygen reduction reaction. Chem. Commun..

[13] Reichenberger S. (2022). Freezing crystallographic
defects into nanoparticles: The development of pulsed laser defect
engineering in liquid (PUDEL). Sci. China: Phys.
Mech. Astron..

[14] Koch N. G., Poschmann M., Heumann S., Puthussery A. J., Čolić V., Barcikowski S., Reichenberger S. (2025). Origin and
role of reactive oxygen species in UV-PUDEL irradiation of platinum
nanoparticles: Effects on surface groups and electrochemical activity. ChemCatChem.

[15] Matten M., Koch N. G., Caidi A., Radev I., Lange T., Barcikowski S., Reichenberger S. (2025). Improved electrochemical activity
of Pt/C catalysts through mild ultraviolet pulsed laser post irradiation. J. Phys. Chem. C.

[16] Feng X., Jiang K., Fan S., Kanan M. W. (2016). A direct
grain-boundary-activity
correlation for CO electroreduction on Cu nanoparticles. ACS Cent. Sci..

[17] Huang W., Johnston-Peck A. C., Wolter T., Yang W. C. D., Xu L., Oh J., Reeves B. A., Zhou C., Holtz M. E., Herzing A. A., Lindenberg A. M., Mavrikakis M., Cargnello M. (2021). Steam-created
grain boundaries for methane C-H activation in palladium catalysts. Science.

[18] Hu M., Poulikakos D., Grigoropoulos C. P., Pan H. (2010). Recrystallization of
picosecond laser-melted ZnO nanoparticles in a liquid: A molecular
dynamics study. J. Chem. Phys..

[19] Shih C.-Y., Shugaev M. V., Wu C., Zhigilei L. V. (2017). Generation of subsurface
voids, incubation effect, and formation of nanoparticles in short
pulse laser interactions with bulk metal targets in liquid: Molecular
dynamics study. J. Phys. Chem. C.

[20] Huang H., Zhigilei L. V. (2022). Computational study
of laser fragmentation in liquid:
Phase explosion, inverse Leidenfrost effect at the nanoscale, and
evaporation in a nanobubble. Sci. China: Phys.
Mech. Astron..

[21] Huang H., Zhigilei L. V. (2021). Atomistic view of
laser fragmentation of gold nanoparticles
in a liquid environment. J. Phys. Chem. C.

[22] Chen C., Zhigilei L. V. (2023). Atomistic modeling
of pulsed laser ablation in liquid:
spatially and time-resolved maps of transient nonequilibrium states
and channels of nanoparticle formation. Appl.
Phys. A.

[23] He N., Hu T., Kang X., Wei S., Wu L., Ye Y., Cai Y., Li P., Liang C. (2025). Laser fragmentation breaks the limitation
of twin-forming ability of fcc metals (Pt/Rh/Pd) with high stacking
fault energy. J. Phys. Chem. C.

[24] Li W., Chen X. (2015). Gold nanoparticles
for photoacoustic imaging. Nanomedicine.

[25] Wilson O. M., Hu X., Cahill D. G., Braun P. V. (2002). Colloidal metal particles as probes
of nanoscale thermal transport in fluids. Phys.
Rev. B.

[26] Ge Z., Cahill D. G., Braun P. V. (2004). AuPd metal
nanoparticles as probes
of nanoscale thermal transport in aqueous solution. J. Phys. Chem. B.

[27] Hartland G. V. (2011). Optical
studies of dynamics in noble metal nanostructures. Chem. Rev..

[28] Metwally K., Mensah S., Baffou G. (2015). Fluence threshold for photothermal
bubble generation using plasmonic nanoparticles. J. Phys. Chem. C.

[29] Stoll T., Maioli P., Crut A., Rodal-Cedeira S., Pastoriza-Santos I., Vallée F., Del Fatti N. (2015). Time-resolved
investigations of the cooling dynamics of metal nanoparticles: impact
of environment. J. Phys. Chem. C.

[30] Schmidt A. J., Alper J. D., Chiesa M., Chen G., Das S. K., Hamad-Schifferli K. (2008). Probing the
gold nanorod-ligand-solvent interface by
plasmonic absorption and thermal decay. J. Phys.
Chem. C.

[31] Alper J., Hamad-Schifferli K. (2010). Effect of
ligands on thermal dissipation from gold
nanorods. Langmuir.

[32] Gonzalez M. G., Acosta E. O., Santiago G. D. (2018). Determination
of the thermal boundary
conductance of gold nanoparticles in aqueous solution using a method
based on nanobubble generation. Appl. Opt..

[33] Plech A., Kotaidis V., Grésillon S., Dahmen C., von Plessen G. (2004). Laser-induced
heating and melting of gold nanoparticles studied by time-resolved
x-ray scattering. Phys. Rev. B.

[34] Dou Y., Zhigilei L. V., Winograd N., Garrison B. J. (2001). Explosive boiling
of water films adjacent to heated surfaces: A microscopic description. J. Phys. Chem. A.

[35] Hu M., Petrova H., Hartland G. V. (2004). Investigation of the properties of
gold nanoparticles in aqueous solution at extremely high lattice temperatures. Chem. Phys. Lett..

[36] Kotaidis V., Dahmen C., von Plessen G., Springer F., Plech A. (2006). Excitation
of nanoscale vapor bubbles at the surface of gold nanoparticles in
water. J. Chem. Phys..

[37] Lapotko D. (2009). Optical excitation
and detection of vapor bubbles around plasmonic nanoparticles. Opt. Express.

[38] Siems A., Weber S. A. L., Boneberg J., Plech A. (2011). Thermodynamics of nanosecond
nanobubble formation at laser-excited metal nanoparticles. New J. Phys..

[39] Katayama T., Setoura K., Werner D., Miyasaka H., Hashimoto S. (2014). Picosecond-to-nanosecond
dynamics of plasmonic nanobubbles from pump-probe spectral measurements
of aqueous colloidal gold nanoparticles. Langmuir.

[40] Moon S., Zhang Q., Xu Z., Huang D., Kim S., Schiffbauer J., Lee E., Luo T. (2021). Plasmonic nanobubbles
– A perspective. J. Phys. Chem. C.

[41] Merabia S., Keblinski P., Joly L., Lewis L. J., Barrat J.-L. (2009). Critical
heat flux around strongly heated nanoparticles. Phys. Rev. E.

[42] Merabia S., Shenogin S., Joly L., Keblinski P., Barrat J.-L. (2009). Heat transfer from nanoparticles: A corresponding state
analysis. Proc. Natl. Acad. Sci. U.S.A..

[43] Nair A. R., Sathian S. P. (2016). Heat transfer across
nanoparticle-liquid interfaces. J. Heat Transfer.

[44] Tascini A. S., Armstrong J., Chiavazzo E., Fasano M., Asinari P., Bresme F. (2017). Thermal transport across nanoparticle–fluid
interfaces: The interplay of interfacial curvature and nanoparticle–fluid
interactions. Phys. Chem. Chem. Phys..

[45] Gutiérrez-Varela O., Merabia S., Santamaria R. (2022). Size-dependent effects of the thermal
transport at gold nanoparticle–water interfaces. J. Chem. Phys..

[46] Wilson B. A., Nielsen S. O., Randrianalisoa J. H., Qin Z. (2022). Curvature and temperature-dependent
thermal interface conductance between nanoscale gold and water. J. Chem. Phys..

[47] Neidhart S. M., Gezelter J. D. (2018). Thermal transport
is influenced by nanoparticle morphology:
A molecular dynamics study. J. Phys. Chem. C.

[48] Jiang M., Olarte-Plata J. D., Bresme F. (2022). Heterogeneous thermal conductance
of nanoparticle–fluid interfaces: An atomistic nodal approach. J. Chem. Phys..

[49] Paniagua-Guerra L. E., Ramos-Alvarado B. (2023). Thermal transport
across flat and curved gold–water
interfaces: Assessing the effects of the interfacial modeling parameters. J. Chem. Phys..

[50] Gutiérrez-Varela O., Lombard J., Biben T., Santamaria R., Merabia S. (2023). Vapor nanobubbles around
heated nanoparticles: Wetting
dependence of the local fluid thermodynamics and kinetics of nucleation. Langmuir.

[51] Chen X., Munjiza A., Zhang K., Wen D. (2014). Molecular
dynamics
simulation of heat transfer from a gold nanoparticle to a water pool. J. Phys. Chem. C.

[52] Shavalier S. A., Gezelter J. D. (2023). Heat transfer in gold interfaces capped with thiolated
polyethylene glycol: A molecular dynamics study. J. Phys. Chem. B.

[53] Baffou G., Rigneault H. (2011). Femtosecond-pulsed optical heating
of gold nanoparticles. Phys. Rev. B.

[54] Pustovalov V. K. (2016). Light-to-heat
conversion and heating of single nanoparticles, their assemblies,
and the surrounding medium under laser pulses. RSC Adv..

[55] Sun J. M., Gerstman B. S., Li B. (2000). Bubble dynamics
and shock waves generated
by laser absorption of a photoacoustic sphere. J. Appl. Phys..

[56] Volkov A. N., Sevilla C., Zhigilei L. V. (2007). Numerical modeling of short pulse
laser interaction with Au nanoparticle surrounded by water. Appl. Surf. Sci..

[57] Pustovalov V. K., Smetannikov A. S., Zharov V. P. (2008). Photothermal and
accompanied phenomena
of selective nanophotothermolysis with gold nanoparticles and laser
pulses. Laser Phys. Lett..

[58] Lombard J., Biben T., Merabia S. (2014). Kinetics of
nanobubble generation
around overheated nanoparticles. Phys. Rev.
Lett..

[59] Lombard J., Biben T., Merabia S. (2017). Threshold
for vapor nanobubble generation
around plasmonic nanoparticles. J. Phys. Chem.
C.

[60] Zhao C., Sun H., Zheng Y., Li S., Yang H. (2023). Promotion of nanobubble
formation around light-induced plasmonic nanoparticles: A molecular
dynamics and continuum modeling comparative study. J. Phys. Chem. C.

[61] Thompson A. P., Aktulga H. M., Berger R., Bolintineanu D. S., Brown W. M., Crozier P. S., in’t Veld P. J., Kohlmeyer A., Moore S. G., Nguyen T. D., Shan R., Stevens M. J., Tranchida J., Trott C., Plimpton S. J. (2022). LAMMPS
- a flexible simulation tool for particle-based materials modeling
at the atomic, meso, and continuum scales. Comput.
Phys. Commun..

[62] Zhukhovitskii D. I., Zhakhovsky V. V. (2020). Thermodynamics and the structure
of clusters in the
dense Au vapor from molecular dynamics simulation. J. Chem. Phys..

[63] Arblaster J. W. (2016). Thermodynamic
properties of gold. J. Phase Equilib. Diffus..

[64] Bhattarai H., Newman K. E., Gezelter J. D. (2020). The role
of polarizability in the
interfacial thermal conductance at the gold-water interface. J. Chem. Phys..

[65] Berendsen H. J. C., Grigera J. R., Straatsma T. P. (1987). The missing
term in effective pair
potentials. J. Phys. Chem. A.

[66] Brovchenko I., Geiger A., Oleinikova A. (2005). Liquid-liquid
phase transitions in
supercooled water studied by computer simulations of various water
models. J. Chem. Phys..

[67] Lemmon, E. W. ; Bell, I. H. ; Huber, M. L. ; McLinden, M. O. Thermophysical Properties of Fluid Systems. In NIST Chemistry WebBook; Linstrom, P. J. ; Mallard, W. G. , Eds.; National Institute of Standards and Technology: Gaithersburg MD, 2023; https://webbook.nist.gov/chemistry/fluid. NIST Standard Reference Database Number 69.

[68] Bedrov D., Smith G. (2000). Thermal conductivity of molecular fluids from molecular dynamics
simulations: Application of a new imposed-flux method. J. Chem. Phys..

[69] Zhang M., Lussetti E., de Souza L. E. S., Müller-Plathe F. (2005). Thermal conductivities
of molecular liquids by reverse nonequilibrium molecular dynamics. J. Phys. Chem. B.

[70] Mao Y., Zhang Y. (2012). Thermal conductivity,
shear viscosity and specific heat of rigid
water models. Chem. Phys. Lett..

[71] Römer F., Lervik A., Bresme F. (2012). Nonequilibrium
molecular dynamics
simulations of the thermal conductivity of water: A systematic investigation
of the SPC/E and TIP4P/2005 models. J. Chem.
Phys..

[72] Ciccotti G., Ryckaert J. P. (1986). Molecular dynamics simulation of rigid molecules. Comput. Phys. Rep..

[73] Hockney, R. W. ; Eastwood, J. W. Computer Simulation Using Particles; Adam Hilger: Bristol, England, 1988.

[74] Hu H., Sun Y. (2012). Effect of nanopatterns
on Kapitza resistance at a water-gold interface
during boiling: A molecular dynamics study. J. Appl. Phys..

[75] Schrader M. E. (1970). Ultrahigh-vacuum
techniques in the measurement of contact angles. II. Water on gold. J. Phys. Chem. A.

[76] Stoddard S. D., Ford J. (1973). Numerical experiments on the stochastic behavior of a Lennard-Jones
gas system. Phys. Rev. A.

[77] Spohr E. (1995). Ion adsorption
on metal surfaces. The role of water-metal interactions. J. Mol. Liq..

[78] De
Anda Villa M., Gaudin J., Amans D., Boudjada F., Bozek J., Evaristo Grisenti R., Lamour E., Laurens G., Macé S., Nicolas C., Papagiannouli I., Patanen M., Prigent C., Robert E., Steydli S., Trassinelli M., Vernhet D., Lévy A. (2019). Assessing
the surface oxidation state of free-standing gold nanoparticles produced
by laser ablation. Langmuir.

[79] Fong Y.-Y., Gascooke J. R., Visser B. R., Harris H. H., Cowie B. C. C., Thomsen L., Metha G. F., Buntine M. A. (2013). Influence of cationic
surfactants on the formation and surface oxidation states of gold
nanoparticles produced via laser ablation. Langmuir.

[80] Schneider T., Stoll E. (1978). Molecular-dynamics
study of a three-dimensional one-component model
for distortive phase transitions. Phys. Rev.
B.

[81] Berendsen H. J. C., Postma J. V., Van Gunsteren W. F., DiNola A. R. H. J., Haak J. R. (1984). Molecular dynamics with coupling
to an external bath. J. Chem. Phys..

[82] Chen G. (2022). Perspectives
on molecular-level understanding of thermophysics of liquids and future
research directions. J. Heat Transfer.

[83] Huber M. L., Perkins R. A., Friend D. G., Sengers J. V., Assael M. J., Metaxa I. N., Miyagawa K., Hellmann R., Vogel E. (2012). New international
formulation for the thermal conductivity of H_2_O. J. Phys. Chem. Ref. Data.

[84] Murad S., Puri I. K. (2008). Thermal transport across nanoscale solid-fluid interfaces. Appl. Phys. Lett..

[85] Cahill D. G., Ford W. K., Goodson K. E., Mahan G. D., Majumdar A., Maris H. J., Merlin R., Phillpot S. R. (2003). Nanoscale thermal
transport. J. Appl. Phys..

[86] Murad S., Puri I. K. (2008). Molecular simulation
of thermal transport across hydrophilic
interfaces. Chem. Phys. Lett..

[87] Alosious S., Kannam S. K., Sathian S. P., Todd B. D. (2019). Prediction of Kapitza
resistance at fluid-solid interfaces. J. Chem.
Phys..

[88] Alexeev D., Chen J., Walther J. H., Giapis K. P., Angelikopoulos P., Koumoutsakos P. (2015). Kapitza resistance between few-layer graphene and water:
liquid layering effects. Nano Lett..

[89] Muscatello J., Chacón E., Tarazona P., Bresme F. (2017). Deconstructing temperature
gradients across fluid interfaces: the structural origin of the thermal
resistance of liquid-vapor interfaces. Phys.
Rev. Lett..

[90] Liang Z., Evans W., Keblinski P. (2013). Equilibrium and nonequilibrium molecular
dynamics simulations of thermal conductance at solid-gas interfaces. Phys. Rev. E.

[91] Giri A., Hopkins P. E. (2016). Analytical model for thermal boundary
conductance and
equilibrium thermal accommodation coefficient at solid/gas interfaces. J. Chem. Phys..

[92] Chen J., Xu X., Zhou J., Li B. (2022). Interfacial thermal resistance: Past,
present, and future. Rev. Mod. Phys..

[93] Wagner W., Pruß A. (2002). The IAPWS
formulation 1995 for the thermodynamic properties
of ordinary water substance for general and scientific use. J. Phys. Chem. Ref. Data.

[94] Stocker K. M., Neidhart S. M., Gezelter J. D. (2016). Interfacial
thermal conductance of
thiolate-protected gold nanospheres. J. Appl.
Phys..

[95] Vega C., de Miguel E. (2007). Surface tension of the most popular models of water
by using the test-area simulation method. J.
Chem. Phys..

[96] Lombard J., Lam J., Detcheverry F., Biben T., Merabia S. (2021). Strong and fast rising
pressure waves emitted by plasmonic vapor nanobubbles. Phys. Rev. Res..

[97] Pham A., Barisik M., Kim B. (2013). Pressure dependence
of Kapitza resistance
at gold/water and silicon/water interfaces. J. Chem. Phys..

[98] Dong M., Xu J., Wang Y., Liu G. (2024). Effect of pressure and surface wettability
on thermal resistance across solid–liquid interface in supercritical
regime. J. Phys. Chem. C.

[99] Anisimov S. I., Kapeliovich B. L., Perel’man T. L. (1974). Electron emission from metal surfaces
exposed to ultrashort laser pulses. Sov. Phys.
JETP.

[100] Balling, P. Laser Coupling and Relaxation of the Absorbed Energy: Metals, Semiconductors, and Dielectrics. In Handbook of Laser Micro- and Nano-Engineering; Sugioka, K. , Ed.; Springer: Cham, Switzerland, 2021; pp 3–59.

[101] Here, the term “lattice temperature” does not imply the preservation of the crystal structure of the nanoparticle but only follows the convention used in the description of the results of TTM calculations, regardless of the phase state of the nanoparticle material.

[102] Reif, F. Fundamentals of Statistical and Thermal Physics; McGraw-Hill: Singapore, 1965.

[103] Tong Z., Li S., Ruan X., Bao H. (2019). Comprehensive
first-principles analysis of phonon thermal conductivity and electron-phonon
coupling in different metals. Phys. Rev. B.

[104] Lin Z., Zhigilei L. V., Celli V. (2008). Electron-phonon
coupling and electron
heat capacity of metals under conditions of strong electron-phonon
nonequilibrium. Phys. Rev. B.

[105] Ivanov D. S., Zhigilei L. V. (2003). Combined atomistic-continuum
modeling
of short-pulse laser melting and disintegration of metal films. Phys. Rev. B.

[106] Sedao X., Shugaev M. V., Wu C., Douillard T., Esnouf C., Maurice C., Reynaud S., Pigeon F., Garrelie F., Zhigilei L. V., Colombier J.-P. (2016). Growth
twinning and generation of high-frequency surface nanostructures in
ultrafast laser-induced transient melting and resolidification. ACS Nano.

[107] He M., Zhigilei L. V. (2024). Multiscale modeling of short pulse laser induced amorphization
of silicon. J. Appl. Phys..

[108] Hu M., Hartland G. V. (2002). Heat dissipation for Au particles in aqueous solution:
Relaxation time versus size. J. Phys. Chem.
B.

[109] Ikeshoji T., Hafskjold B. (1994). Non-equilibrium
molecular dynamics
calculation of heat conduction in liquid and through liquid-gas interface. Mol. Phys..

[110] Zhigilei L. V., Garrison B. (1998). Microscopic simulation of short pulse
laser damage of melanin particles. Proc. SPIE.

[111] Zhigilei L. V., Garrison B. J. (1998). Pressure waves in microscopic simulations
of laser ablation. MRS Proc..

[112] Plech A., Tack M., Huang H., Arefev M., Ziefuss A. R., Levantino M., Karadas H., Chen C., Zhigilei L. V., Reichenberger S. (2024). Physical regimes and mechanisms of
picosecond laser fragmentation of gold nanoparticles in water from
X-ray probing and atomistic simulations. ACS
Nano.

[113] Arefev M. I., Shugaev M. V., Zhigilei L. V. (2022). Kinetics of laser-induced
melting of thin gold film: How slow can it get?. Sci. Adv..

[114] Luo S.-N., Ahrens T. J., Çağın T., Strachan A., Goddard W. A., Swift D. C. (2003). Maximum
superheating and undercooling: Systematics, molecular dynamics simulations,
and dynamic experiments. Phys. Rev. B.

[115] Chan W.-L., Averback R. S., Cahill D. G., Ashkenazy Y. (2009). Solidification
velocities in deeply undercooled silver. Phys.
Rev. Lett..

[116] Hoyt J. J., Asta M. (2002). Atomistic computation of liquid diffusivity,
solid-liquid inter- facial free energy, and kinetic coefficient in
Au and Ag. Phys. Rev. B.

[117] He M., Karim E. T., Shugaev M. V., Zhigilei L. V. (2021). Atomistic simulation
of the generation of vacancies in rapid crystallization of metals. Acta Mater..

[118] Honeycutt J. D., Andersen H. C. (1987). Molecular dynamics study of melting
and freezing of small Lennard-Jones clusters. J. Phys. Chem. A.

[119] Stukowski A. (2010). Visualization and analysis of atomistic simulation
data with OVITO—the Open Visualization Tool. Model. Simul. Mater. Sci. Eng..

[120] Zhigilei L. V., Ivanov D. S. (2005). Channels of energy
redistribution
in short-pulse laser interactions with metal targets. Appl. Surf. Sci..

[121] Ivanov D. S., Zhigilei L. V. (2003). Effect of pressure relaxation on
the mechanisms of short-pulse laser melting. Phys. Rev. Lett..

[122] Sasikumar K., Keblinski P. (2014). Molecular dynamics investigation
of nanoscale cavitation dynamics. J. Chem. Phys..

